# The effect of synthesis conditions and process parameters on aerogel properties

**DOI:** 10.3389/fchem.2023.1294520

**Published:** 2023-10-23

**Authors:** Ozge Payanda Konuk, Ala A. A. M. Alsuhile, Hamed Yousefzadeh, Zeynep Ulker, Selmi E. Bozbag, C. A. García-González, I. Smirnova, Can Erkey

**Affiliations:** ^1^ Department of Materials Science and Engineering, Koç University, Istanbul, Türkiye; ^2^ Department of Chemical and Biological Engineering, Koç University, Istanbul, Türkiye; ^3^ Department of Chemical Engineering, Yeditepe University, Atasehir, Istanbul, Türkiye; ^4^ School of Pharmacy, Altinbas University, Istanbul, Türkiye; ^5^ Departamento de Farmacología, Farmacia Y Tecnología Farmacéutica, I+D Farma (GI-1645), Faculty of Pharmacy, Instituto de Materiales (iMATUS) and Health Research Institute of Santiago de Compostela (IDIS), Universidade de Santiago de Compostela, Santiago de Compostela, Spain; ^6^ Institute of Thermal Separation Processes, Hamburg University of Technology, Hamburg, Germany; ^7^ Koç University Tüpraş Energy Center (KUTEM), Koç University, Istanbul, Türkiye

**Keywords:** aerogel, supercritical, sol-gel, aging, solvent exchange, silica, carbon, polysaccharide

## Abstract

Aerogels are remarkable nanoporous materials with unique properties such as low density, high porosity, high specific surface area, and interconnected pore networks. In addition, their ability to be synthesized from various precursors such as inorganics, organics, or hybrid, and the tunability of their properties make them very attractive for many applications such as adsorption, thermal insulation, catalysts, tissue engineering, and drug delivery. The physical and chemical properties and pore structure of aerogels are crucial in determining their application areas. Moreover, it is possible to tailor the aerogel properties to meet the specific requirements of each application. This review presents a comprehensive review of synthesis conditions and process parameters in tailoring aerogel properties. The effective parameters from the dissolution of the precursor step to the supercritical drying step, including the carbonization process for carbon aerogels, are investigated from the studies reported in the literature.

## 1 Introduction

Aerogels are solid, open porous networks formed by replacing the liquid phase in a gel with the gas phase. Inorganic and organic aerogels were first synthesized by Samuel Kistler in 1932 (Kistler, 1928). Since then, aerogels from various precursors have been studied. They can be classified based on their chemical compositions as inorganic (silica, alumina, titania), organic (resorcinol formaldehyde, cellulose, pectin), carbon (via pyrolysis of organic aerogels), and hybrid/composite aerogels. Aerogels have low density, high porosity, high surface area, and open porous structure (Hüsing and Schubert, 1998; Smirnova and Gurikov, 2018). These distinctive properties make them very attractive for many applications such as thermal insulation (Jones, 2006; Baetens et al., 2011), acoustic insulation (Feng et al., 2016), solar systems (Strobach et al., 2017), drug delivery (Ulker and Erkey, 2014; Ulker and Erkey, 2017), tissue engineering (Mirtaghavi et al., 2020), catalysts and supports (Bozbag et al., 2012; Barım et al., 2017; Sarac Oztuna et al., 2017; Barim et al., 2018; Ünsal et al., 2019; Lee and Park, 2020; Barim et al., 2022; Yousefzadeh et al., 2022), adsorption (Jayne et al., 2005; Maleki, 2016; Anas et al., 2017), sensors (Yang et al., 2019), and energy conversion and storage applications (Biener et al., 2011; Alwin and Sahaya Shajan, 2020).

Briefly, to produce an aerogel, first of all, a gel is formed. After that, the gel is aged and subjected to a series of solvent exchanges with a suitable solvent. Finally, the obtained wet gel is dried to obtain an aerogel. To tailor and improve the aerogel properties for a particular application, it is essential to understand the mechanisms behind the synthesis routes and effective parameters during processing.

This review gives an overview of how the aerogel properties, such as bulk density, porosity, specific surface area, and volumetric shrinkage, vary with synthesis conditions and process parameters. The review also includes models for each step that may be used for scale-up.

Aerogel synthesis routes and the impact of precursors, pore-filling solvents, catalyst and pH, surfactant, and gelation conditions are discussed in the section titled “Solution Preparation and Gelation”. The aging mechanism, various factors that affect aerogel structure during the aging step, and models for aging are explained in the section titled “Aging”. The solvent exchange’s necessity, the solvent choice’s importance, and the effect of concentration gradient, duration, and temperature on aerogel properties are given in the section titled “Solvent Exchange”. The supercritical drying of gels and the affecting parameters and scale-up models are examined in the section titled “Supercritical Drying”. Carbonization process conditions and their impacts are given in the section titled “Carbonization”.

## 2 Solution Preparation and Gelation

**FIGURE 1:**
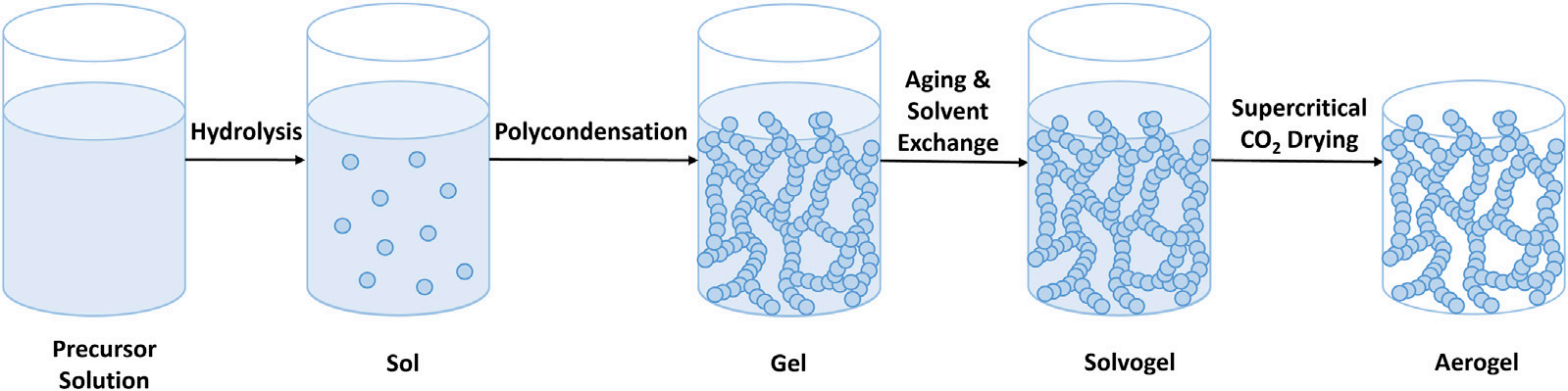
Hydrolysis and polycondensation sol-gel processing.

The network structure of the gels, and therefore the properties of the aerogels, are affected by the properties of precursors and the reaction parameters of the gelation processes. Gels are prepared by sol-gel processing, and the type of sol-gel processing can be divided into two main sub-groups. One method is hydrolysis, followed by polycondensation reactions, which starts with the dissolution of a monomer in a solvent (Ratke and Gurikov, 2021a). All of the inorganic aerogels (such as silica, alumina, and zirconium) and some organic aerogels, such as resorcinol–formaldehyde (RF) and melamine-formaldehyde (MF) aerogels, are prepared with this sol-gel processing (Figure 1) (Pekala, 1989; Mulik et al., 2011; Pierre et al., 2011).

**FIGURE 2:**
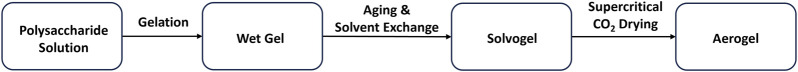
General synthesis of biopolymer aerogels.

The other method (Figure 2) is first dissolving the polymer in a solution and then regenerating the polymer network with physical crosslinking (such as temperature or pH) or chemical crosslinking. This method is generally used for biopolymer aerogels such as cellulose, alginate, starch, carrageenan, and pectin (Ulker and Erkey, 2014; Ganesan et al., 2018; Ratke and Gurikov, 2021a).

### 2.1 Effect of precursor type and concentration

The molecular weight, the molecular structure, and the concentration of the precursor used have an impact on the aerogel structure and density. Aerogels with low precursor concentrations are generally unable to preserve the structure and show higher volumetric shrinkages during solvent exchange and supercritical drying. On the other hand, higher precursor concentrations lead to denser and more interconnected structures, which also affect the porosity and the specific surface area of aerogels.

Venkateswara Rao et al. investigated the different molar ratios of H_2_O/Na_2_SiO_3_ between 83.3 and 333.3 for the synthesis of silica aerogels (Shewale et al., 2009). The experiments showed that decreasing silicate concentration increased the gelation time from 1 min to 6 h. This was ascribed to the lower collision frequency due to the increased distance between the silica particles. Additionally, increasing the molar ratio of H_2_O/Na_2_SiO_3_ up to 166.6 resulted in larger colloidal silica particles; hence, larger pore size, lower volumetric shrinkage, and lower density. However, they also emphasized that with the further decreasing concentration of Na_2_SiO_3_, the bulk density of the aerogel increased, which can be explained by the volumetric shrinkage due to the fragile network.

Bedilo and Klabunde found similar results for zirconia aerogels. They indicated that the pore sizes and the pore volumes decreased with the increasing precursor concentration in zirconia aerogels (Bedilo and Klabunde, 1997). This is due to the fact that as the precursor concentration increases, the polycondensation reaction rate increases, which leads to smaller pore size and greater bulk density. Moreover, the specific surface area of the zirconia aerogels increased from 325 m^2^/g to 450 m^2^/g, with the precursor concentration increasing from 0.25 M to 1 M.

Méndez et al. studied the effect of molecular weight, polymer chemistry, and the pectin concentration on pectin aerogel structure (Méndez et al., 2023). They prepared pectin aerogels using pectin concentrations of 2 and 4 wt% in de-mineralized water from 4 different pectins: a citrus pectin containing 5% methoxy (C1), a low molecular weight pectin (C2), a high molecular weight pectin (C3) and in-house produced watermelon rind pectin (WRP). They indicated that the precursor’s source affected the hydrogel, alcogel, and aerogels’ morphology and shape. The commercial pectin aerogels were more transparent due to their more homogenous composition. In addition, C2 aerogels showed non-uniform shapes, which was ascribed to their low molecular weight and low viscosity. Aerogels with low pectin concentrations showed critical volumetric shrinkage in both solvent exchange and supercritical drying processes. However, increasing pectin concentration decreased the volumetric shrinkage. They also added that the chemical composition and the chemical structure of the precursor affect the volumetric shrinkage of the aerogels. Aerogels prepared from a lower degree of esterification and higher homogalacturonan content showed the lowest volumetric shrinkage, which was attributed to the more robust gel network. An interesting result was that increasing the concentration of high molecular weight pectin aerogel (C3) led to higher volumetric shrinkage. The authors attributed this to the greater degree of branching, which was also consistent with previous studies. The specific surface areas of pectin aerogels were between 469 and 585 m^2^/g. Contrary to expectations and previous studies, the specific surface area of the aerogels did not significantly increase with the increasing pectin concentration. A possible explanation for this might be the constant 0.5% (w/w) CaCl_2_ concentration used as a crosslinker. As expected, higher volumetric shrinkages caused higher bulk densities. In addition, lower volumetric shrinkages caused lower bulk densities, which led to higher pore diameters and pore volumes ranging from 202 to 137 Å and 2.37–8.65 cm^3^/g, respectively.

Gavillon and Budtova prepared various cellulose aerogels, also called Aerocellulose, using several cellulose types with different origins and degrees of polymerization (DP) (Gavillon and Budtova, 2008). They pointed out that the source of the cellulose does not have a significant impact on aerogel porosity. They chose Avicel PH-101 microcrystalline cellulose (DP = 180) as a precursor to investigate the effect of cellulose concentration on aerogel pore properties. They prepared the aerocellulose using cellulose concentrations of 5, 6, and 7 wt% in NaOH/water solutions. With the increasing cellulose concentration, the mean pore diameter, the total cumulative pore volume, and the specific surface area decreased from 0.9 µ to 0.7 µ, from 6.6 g/cm^3^ to 6 g/cm^3,^ and from 240 m^2^/g to 200 m^2^/g, respectively. They also examined the effect of initial cellulose concentration on the mechanical properties of aerogels. For the three types of cellulose, the results showed that increasing the precursor concentration increased the gel strength. This may be attributed to the more robust network structure due to the increased cellulose chain interactions.

Jin et al. prepared nanofibrillar cellulose aerogels by dissolving cellulose powder in calcium thiocyanate tetrahydrate solution (Jin et al., 2004). The prepared cellulose solutions had low cellulose content (0.five to three wt%), leading to low density aerogels. The results showed that increasing the cellulose concentration increased the aerogel density almost proportionally, as expected. It was also stated that the mechanical strength of the nanofibrillar cellulose aerogels increased from 0.5 N/cm^2^ to 11 N/cm^2^. However, it was deduced that elongation at break was independent of the initial precursor content. Jin and co-workers also investigated the effect of cellulose content on specific surface areas. According to the results, increasing cellulose concentration from 0.5 to 3 wt% increased the surface area from 160 m^2^/g to 190 m^2^/g.

### 2.2 Effect of pore liquid/type of the solvent

During the sol-gel processes, the type of solvent used also has an impact on aerogel properties like surface area, porosity, morphology, and mechanical stability. The precursor should be soluble in the solvent at the utilized concentrations to prepare homogeneous gels. In addition, the solvent’s polarity and viscosity may affect the rates of network forming reactions (Gurav et al., 2010).

Alkoxides are not miscible in water. Thus, in order to be able to form a sol, alkoxides are dissolved in water/alcohol mixtures. Mostly, methanol and ethanol are used for TMOS and TEOS, respectively. In this case, the dilution ratio of water to alkoxide and alcohol to alkoxide affects the sol-gel reactions (Ratke and Gurikov, 2021b). Rao and Bhagat pointed out that increasing the EtOH to TEOS ratio led to a decrease in the bulk density and the volumetric shrinkage and an increase in the optical transmission of the aerogels (Rao and Bhagat, 2004).

Rao et al. also investigated six different solvents: methanol, ethanol, propanol, butanol, acetone, and acetonitrile. Results showed that the chain length and branching of the solvent were directly proportional to the gelation time, and the solvent also affected the density, specific surface area, pore volume, and porosity of the aerogels (Venkateswara Rao et al., 1994). Aerogels prepared using methanol had the lowest density (0.050 g/cm^3^), the highest specific surface area (1050 m^2^/g), the highest pore volume (19.32 cm^3^/g), and the highest porosity (97.5%). Alcohols having smaller alkyl groups exhibited smaller steric hindrance; hence, smaller pore sizes and more transparent aerogels were obtained with methanol.

Pircher et al. studied the impact of different solvent systems on the properties of cellulose aerogels from cotton linters (Pircher et al., 2016). For this purpose, four solvent systems, including tetrabutylammonium fluoride and DMSO (TBAF/DMSO), 1-ethyl-3-methyl-1H-imidazolium acetate and DMSO ([EMIm][OAc]/DMSO), calcium thiocyanate octahydrate and lithium chloride (CTO/LiCl), and molten N-methylmorpholine-N-oxide monohydrate (NMMO^.^H_2_O) were used. The results showed that aerogel properties were greatly affected by the phase separation upon standing, cooling, solidification, or addition of a solvent in which cellulose was not soluble. These differences were attributed to distinct mechanisms of cellulose self-assembly at the supramolecular and nanostructural levels, resulting in varying crystallinity, fibril diameter, fractal dimension, and skeletal density. Cellulose aerogels from the CTO solvent system showed the highest crystallinity. Aerogels with larger fibril diameters, higher crystallinity, and increased skeletal density displayed better shape preservation and mechanical stability but had slightly reduced specific surface areas. Notably, aerogels prepared using TBAF/DMSO mixtures showed amorphous yet highly rigid properties, deviating from the observed trends, likely due to their unique homogeneous and nanostructured morphology.

### 2.3 Effect of catalyst type, catalyst concentration, and pH

Catalysts are commonly used to control the hydrolysis and condensation reaction rate, which leads to different microstructures. During the preparation of inorganic aerogels, the hydrolysis reaction kinetics can be accelerated by adding an acid or a base catalyst, and the condensation reaction kinetics can be accelerated by adding a base catalyst (Ulker and Erkey, 2014). Indeed, the ultimate structure of hydrolyzed silica is influenced by the pH level of the solution. Silica particles rearrange into a linear chain structure with limited crosslinking when the pH is low. This gives rise to a highly porous and weak gel structure and may result in redispersion in the solution. At higher pHs, crosslinking between the chains increases, and the network becomes more branched, which leads to a higher density and stronger gel structure (Brinker et al., 1982; Venkateswara Rao et al., 1994; Gurav et al., 2010).

Rao and Bhagat investigated the effects of acid, base, and acid-base catalysts (two-step synthesis) on the properties of TEOS-based silica aerogels (Rao and Bhagat, 2004). The two-step synthesis method was used to control the polymerization of silica. Oxalic acid and NH_4_OH were used as acid and base catalysts, respectively. They indicated that as the base concentration increased, the gelation time decreased from 10 min to 2 min. This stemmed from the increase in condensation reaction rate due to the increase in base concentration. They also added that the gelation time lasted at least 3 days without NH_4_OH catalyst. Up to 1 M base catalyst concentration, densities of the aerogels decreased. However, as the concentration increased further, the aerogel density began to increase due to the weak structure that led to volumetric shrinkage. The authors attributed this to the small particle sizes as a consequence of the sudden condensation reaction. They also investigated the effect of acid concentration by fixing the base concentration to 1 M. It was seen that the gelation time decreased from 30 min to 10 min as the acid concentration increased. They explained that this may be because the hydrolysis reaction rate increases with the increase of acid catalyst concentration, leading to faster condensation with the addition of base. In addition, concentrations higher than 0.001 M led to cracks and opacity in aerogels. Rao and Bhagat also examined the effect of time intervals before adding a base catalyst. It was seen that aerogels with the 24 h time interval gelled in 30 min, and they had low volumetric shrinkage (17.48%), low density (0.1540 g/cm^3^), high porosity (91.89%) and an optical transmittance of 64%.

Tamon et al. prepared silica aerogels using the sol-gel polymerization of tetraethylorthosilicate (TEOS) using HCl and NH_3_ as hydrolysis and condensation catalysts, respectively (Tamon et al., 1998). They examined the various mole ratios of [HCl]/[TEOS] such as $3.45\times 10^{-5}$ , 4 $.30\times 10^{-5}$ , $5.30\times 10^{-5}$ , and $6.56\times 10^{-5}$ and also various mole ratios of [NH_3_]/[TEOS] such as $2.96\times 10^{-3}$ , $4.98\times 10^{-3}$ , $7\times 10^{-3}$ , and $9.02\times 10^{-3}$ . They pointed out that increasing the hydrolysis time from 12 to 77 min and increasing the [HCl]/[TEOS] ratio decreased the gelation time. In addition, an increase in the hydrolysis time led to a higher specific surface area from 440 to 770 m^2^/g, higher mesopore volume from 0.5 to 4.2 cm^3^/g, and higher bulk density from 0.125 to 0.152 g/cm^3^.

Resorcinol–formaldehyde (RF) aerogels are also synthesized by the polycondensation of resorcinol (R) and formaldehyde (F) with acid or base catalysts. Less branched structures are obtained at low pH values, which results in larger polymer particles. On the other hand, at higher pH values, highly crosslinked and branched structures are obtained, which causes a more interconnected network with smaller polymer particles. Moreover, as a consequence of smaller particles and interconnected networks, the specific surface area of the aerogel increases (Pekala and Schaefer, 1993; Horikawa et al., 2004; Feng et al., 2008).

Horikawa et al. prepared RF aerogels using four different base catalysts, which were potassium carbonate (K_2_CO_3_), sodium carbonate (Na_2_CO_3_), potassium hydrogen carbonate (KHCO_3_), and sodium hydrogen carbonate (NaHCO_3_) with different resorcinol/catalyst (R/C) ratios (Horikawa et al., 2004). They pointed out that although micropore volumes were not significantly affected by the type and the concentration of the catalysts, the mesopore volumes and the pore size distributions were affected.

The influence of pH on pectin was also examined. According to Groult et al., pectin has a high sensitivity to pH, resulting in sample shrinkage and density increase at basic conditions, and strong and stable gels can be obtained in acidic conditions (Groult et al., 2021).

### 2.4 Effect of surfactants

Surfactants are generally used to increase the aerogel porosity. For this purpose, Gavillon and Budtova incorporated a non-ionic surfactant Simulsol SL8 at concentrations of 0.1%, 0.5%, and 1% by weight. During the solution preparation, they observed air bubbles due to the presence of surfactant, and these air bubbles trapped in the gels during gelation processes, leading to large pores in the final structure of aerogels. They confirmed the scanning electron microscopy images with mercury intrusion and nitrogen absorption methods. As the surfactant concentration increased from 0% to 1%, the pore diameter and the total pore volume increased from 0.9 µm to 47.5 µm and 6.6 cm^3^/g to 9.6 cm^3^/g, respectively. Consequently, the density of the aerogels decreased by 30% and 60% with the addition of 0.5% and 1% of surfactant, respectively (Gavillon and Budtova, 2008).

Jung et al. prepared alumina aerogels with three types of surfactants: Triton X-100 (non-ionic), cetyltrimethylammonium bromide (cationic), and sodium dodecyl sulfate (anionic) (Jung et al., 2023). The results showed that the type and concentration of the surfactant had an impact on the surface area and pore volume. At critical micelle concentration (CMC), all aerogels with different surfactant types showed the smallest specific surface area. However, above CMC, the specific surface area of the aerogels increased with increasing micelle formation. Regardless of the surfactant type, pore volumes were larger than the pristine alumina aerogels. The pore volume at CMC was smaller than that under and over CMC. When non-ionic and cationic surfactants were used, pore diameters were similar to pristine alumina aerogels (3.42 nm). However, the addition of anionic surfactant caused an increase in the pore diameter (3.42–7.83 nm).

### 2.5 Effect of gelation temperature and time

Although gelation temperature impacts the gelation time, it does not significantly affect the pore properties of aerogels.

The studies of Gavillon and Budtova showed that the selected gelation conditions (25 °C for 24 h and 50 °C for 2 h) did not significantly affect aerogel structure (Gavillon and Budtova, 2008).

Hegde et al. investigated the influence of gelation temperature on TEOS-based aerogels by changing the temperature from 26°C to 70°C (Hegde and Rao, 2006). At 50°C, the gelation temperature reduced from three and a half days to less than a day due to the faster kinetics. However, aerogels produced at higher than 50 °C suffered volumetric shrinkage due to the solvent’s expulsion from the gel.

Wiener et al. investigated the gelation and aging temperature of the RF aerogels (Wiener et al., 2004). The findings showed that increasing gelation temperature led to a decrease in gelation time from 3 days to 1 day. However, the aerogel pore sizes were slightly reduced. Tamon and Ishizaka also found similar results for the temperature range of 25°C–50°C (Tamon and Ishizaka, 2000). They stated that the change in the temperature did not affect the pore size distribution significantly.

### 2.6 Models for gelation

Studies on computational modeling of aerogel structures and their properties has significantly increased since models may enablee engineering of aerogels and enable us to predict aerogel properties before synthesizing them in the laboratory. The modeling approaches of aerogels depend on their morphology, which can be classified as particle-aggregated and fibrillar (Rege, 2023). Inorganic aerogels and some of the organic aerogels, such as RF, MF, and carbon aerogels, show particle-aggregated morphology (Mulik et al., 2007; Sinkó, 2010). On the other hand, biopolymer aerogels, in particular polysaccharides, show fibrillar morphology (Gavillon and Budtova, 2008).

#### 2.6.1 Particle-aggregated aerogels

Although particle-aggregated aerogels have similar network structures, they exhibit different characteristics. For instance, silica aerogels have fractal morphology due to the formation of bonds from nucleation and growth. Fractal aerogels are synthesized by the dissolution of precursor in the solution, and during this process, particles undergo Brownian motion, which means particles move randomly. To model these aerogels, diffusion-limited aggregation (DLA) (Witten and Sander, 1981) and, in particular, diffusion-limited cluster-cluster aggregation (DLCA) approach is mostly used (Meakin, 1984; Hasmy et al., 1994). As an alternative approach, reaction-limited cluster-cluster aggregation (RLCA) is used in which it is assumed that not every collision may form aggregation (Meakin and Family, 1988). Moreover, the ballistic aggregation (BA) approach was also studied, where a particle moves in linear motion before the collision (Grzegorczyk et al., 2004).

On the other hand, aerogels such as RF or MF have non-fractal morphology, which stems from microphase separation. They do not exhibit self-similar patterns. The first study on the non-fractal aerogel model was Gaussian random fields (GRF) approach by Roberts (Roberts, 1997); however, the calculated surface areas were higher than the experimental ones. Therefore, Gommes and Roberts (2008) modified the GRF model, which resulted in a more accurate network structure. Another model for non-fractal aerogels is the polymerization-induced phase separation (PIPS) approach. Wang et al. developed a model for porous microstructures of membranes which is based on Cahn–Hilliard–Navier–Stokes method with Flory-Huggins theory (Wang F. et al., 2020).

#### 2.6.2 Fibrillar aerogels

As mentioned earlier, biopolymer aerogels exhibit fibrillar morphology, which results from aggregation of polymer chains and physical and chemical crosslinking between these chains. To understand the network morphology of fibrillar aerogels, Depta et al. developed a model for Ca-alginates, which was a discrete element method (DEM) based on Langevin dynamics simulation approach. Furthermore, they investigated the effect of different parameters such as precursor concentration, ion concentration, molecular weight, and alginate composition on aerogels, and it was seen that the proposed model agreed with the experimental data (Depta et al., 2022). Another approach to model fibrillar aerogels is Laguerre-Voronoi tessellation, the modified version of Voronoi tessellation used to describe the microstructures of cellular foams. Besides being a faster approach, it is also possible to determine the mechanical and thermal properties of aerogels with the addition of the finite element method (Voronoi, 1908; Chandrasekaran et al., 2021).

#### 2.6.3 Estimation of the gelation time

The estimation of the gelation time can be achieved by Family-Viscek scaling after obtaining the gelation model (Vicsek, 1992). Interestingly, few studies have examined the size dependence of gelation time. For instance, Anglaret et al. (1995) investigated the gelation time as a function of different height to diameter ratios of neutral and weakly base-catalyzed TMOS gels. According to their results, neutrally catalyzed gels were size-independent. Moreover, the gelation time of the weak base-catalyzed gels increased with the increasing size. In addition, they created a model known as the fluctuating bond aggregation (FBA) to clarify their results. In addition, Ratke and Hajduk investigated the size effect of RF gels on gelation time by preparing them in different volumes (Ratke and Hajduk, 2015).

## 3 Aging

Aging can be described as the changes associated with the structure and properties of the gel when it is maintained in its pore liquid. This is due to the fact that upon gelation, precursors might not have been used up completely. Mechanisms, including Ostwald ripening, coarsening, sintering, and syneresis, as described in (67), allow the network to be modified depending on parameters such as concentration, pH, temperature, and time (Ahmad et al., 2023). For silica gels, the presence of remaining Si-OR and Si-OH groups in the gel network, along with unreacted monomers in the pores, makes the reactions continue even though the gelation has occurred. Gels which were catalyzed by bases can be aged for a few days to weeks in water/alcohol solutions with concentrations similar to that of the sol at pH 8–9 (Hench and West, 1990). Native silica aerogels are usually brittle because they have relatively open structures with minimal siloxane linkages. One sophisticated strategy for strengthening the solid skeleton of a silica gel is to increase the number of siloxane bonds connecting the secondary particles through aging (Hæreid et al., 1995; Strøm et al., 2007). During the preparation of the aerogel, the purpose of aging is usually to render a stronger gel network to avoid shrinkage during drying. Washing in the H_2_O/ethanol mixture increased the liquid permeability of the solid part of the gel by a dissolution reprecipitation process for silica. On the other hand, the strength and stiffness of the alcogel are increased when aged in siloxane solution by the addition of available monomers to the silica network and by increasing the cross-linking of siloxane (Soleimani Dorcheh and Abbasi, 2008). Aging usually changes the physical properties of the gel, an example of which is the mechanical properties. For example, the rupture modulus of silica gel depends on aging time and temperature and could increase to reach 40 Pa after 40 days inside the main solution at 105°C (Izadi et al., 2023). However, one has to take into account the effects of aging on other properties as well. For example, the lower thermal conductivity of a silica aerogel could be considered as a result of short aging to limit the growth of thick inter-particle necks and the concomitant increase in thermal conductivity. In addition, shorter aging results in (slightly) larger pore sizes and may reduce the probability of connecting dangling particle chains to the rest of the network (Iswar et al., 2021). Early studies cited in (Hench and West, 1990) showed that formed silica gel structure can be modified in the wet state via treatment to make the structure stronger without significant changes in the pore structure or enlarge the pore size and decrease the surface area via dissolution and redeposition, which results in the coarsening of the gel. Therefore, understanding, careful manipulation, and optimization of aging conditions are paramount to obtaining aerogels with desired properties.

**FIGURE 3:**
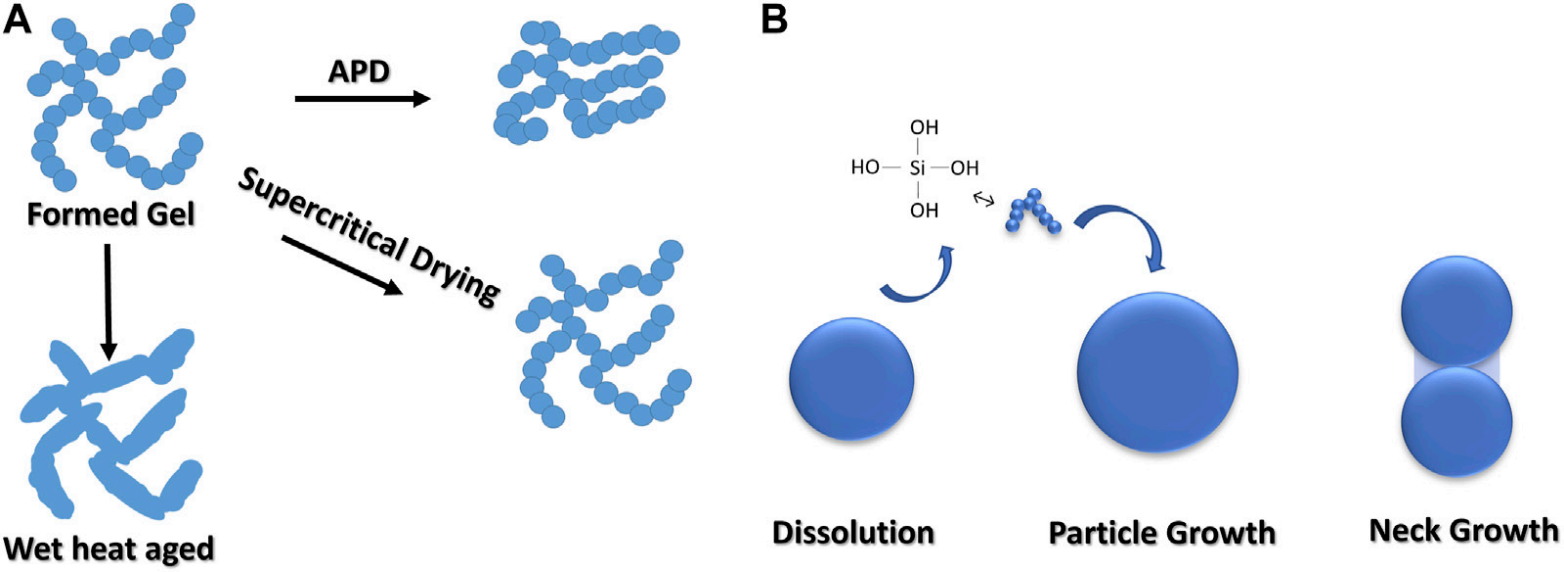
Representation of phenomena occurring during aging **(A)** Formed gel and the effect of drying along with the illustration of consequence of wet heat aging (which results in little shrinkage upon drying). Adapted with permission from (Hench and West, 1990) Copyright 1990 American Chemical Society. **(B)** Particle growth mechanism during aging according to Oswald ripening (left) and neck growth (right). Adapted with permission from (Li et al., 2023a) Copyright 2023 Taylor and Francis Group.

The aforementioned mechanisms might lead to two different phenomena during the aging of the gel, which leads to the modification of its structure and properties. For silica gels, these are (Soleimani Dorcheh and Abbasi, 2008): Growth of the necks between particles from reprecipitation of silica dissolved from particle surface onto necks between particles and dissolution of smaller particles and precipitation onto larger ones. These two processes have different rates while operating in tandem (Strøm et al., 2007). Processes occurring during aging are illustrated in Figure 3. Synthesized gels are often fragile and tend to get more mechanically stable with time when they are kept in their mother liquor. During aging, interparticle necks in the colloidal particles are usually strengthened, which are the points of contact at the time of gelation. Based on the conditions, aging could be accompanied by coarsening and microscopic phase separation of the gel phase. Once the gelation has occurred, residual soluble silicates in the mother liquor deposit. With time, soluble silicates no longer come from the pore liquid but are dissolved from the gel structure. Aging with prolonged times can be described by the Ostwald ripening, where soluble species are transferred between solid and liquid phases, which results in a continuous dissolution-precipitation process. Solubility is said to be the driving force of such a process for the surfaces with different curvatures, which is usually described by the Kelvin equation (Soleimani Dorcheh and Abbasi, 2008). While small particles within the gel network or colloidal particles have a considerable surface free energy, which helps dissolution, interparticle necks have large negative curvatures and lower surface free energy, which ease reprecipitation.

### 3.1 Effects of various factors employed in the aging step

There is a wide variety of studies reporting the effects of the addition of monomers, catalysts, concentrations, pH, and the use of different solvents, along with the effects of the time and temperature of the aging step on the properties of the aerogels. These reports are summarized in this section.

In the aging process for base-catalyzed gels, soaking the gel in an alcohol and water mixture of equal proportions to the original sol at a pH of eight to nine is a standard practice. This step can take place at room temperature (Einarsrud et al., 2001; He et al., 2014). According to Hdach et al. (1990), the influence of pH on densification can be understood in terms of competition between polycondensation, which results in shrinkage and dissolution-reprecipitation, which increases the rigidity of the network. While at low pH, precipitation dominates, at high pH, dissolution-reprecipitation stiffens the gel network and reduces the possibility of shrinkage.

As previously mentioned, aging of the wet gel leads to dissolution and reprecipitation of silica monomers to the gel structure and results in stronger gels. Along this line, Smitha et al. (Smitha et al., 2006) investigated the effect of concentration of the aging solution and duration of aging on the pore characteristics. During aging, new monomers are added to the already formed network, and the precipitation of these monomers increases alcogel strength and stiffness. The specific surface area, pore size, and pore volume increased with increasing TEOS concentration in the aging solution. With an 80% tetraethoxysilane (TEOS) in isopropanol at 50°C, a surface area of 1098 m^2^/g, pore volume of 1.3 cm^3^/g, and an average pore size of 47.7 Å were obtained. Time of aging also had a similar effect on surface area and pore volume as the concentration of aging solution; that is, with increasing aging time, there was an increasing trend; however, after 48h, they all decreased to a certain degree. They also showed that the bulk density and linear drying shrinkage of the aerogel decreased to a certain degree with an increase in the concentration of TEOS and aging time during aging and remained constant upon a further increase in concentration and aging time. Einarsrud et al. (2001) prepared gels from a polyethoxydisiloxane precursor using HF as a catalyst. During washing with water solution, a significant increase in permeability of the gels was observed, showing that dissolution-reprecipitation occurred. After washing, gels were further soaked with a solution of polyethoxydisiloxane, which resulted in the formation of stronger and stiffer gels. While permeability also increased with aging, shear modulus and modulus of rupture decreased with increased aging cycles. It was observed that washing and aging treatments increased both the cluster and particle dimensions based on small angle X-ray scattering (SAXS) data, with this effect being more prominent at higher temperatures. In order to improve the mechanical properties of aerogels, Strøm et al. (2007) performed different aging methods for the wet gels synthesized from polyethoxydisiloxane precursor with HF catalyst, which were aged in sealed mold, aged in solvent and aged in simulated pore liquid (a solvent (ethanol or ethyl acetoacetate) with small amounts of water and HF resembling the mother liquor). All of these aging methods resulted in stronger and stiffer gels with maximum mechanical properties achieved after a certain time. The trend for surface area with respect to aging time was a decreasing one due to the filling of the necks pore coarsening during aging; however, the extent of the decrease depended on the solvent used. The decrease in surface area corresponded to the change in shrinkage of the wet gels during aging, with a much more rapid decrease in surface area for gels aged in simulated pore liquids. The driving force for the mass transport in both mechanisms was the dependence of silica solubility on the curvature, which meant that the change in surface area of the aerogel could be correlated to the increase in neck size during aging and hence the shear modulus of the wet gel. Therefore, they concluded that the surface area is an important parameter that determines the strength and stiffness of a wet gel.

Estella et al. (2007) investigated the effect of several aging conditions on the structure and porosity of the gels. Gels were aged in ethanol or aqueous NH_3_. It was found that aging in ethanol rendered aerogels with higher surface area (549 vs. 337–381 m^2^/g, for ethanol and NH_3_(aq), respectively) and pore volume (0.149 vs. 0.111–0.097 cm^3^/g, for ethanol and NH_3_(aq), respectively) than the ones aged in aqueous NH_3_. Omranpour et al. (Omranpour and Motahari, 2013) investigated the parameters that enhanced the mechanical properties of silica aerogel during aging. Silica aerogel was synthesized by TEOS, water, methanol, and NH_4_F. Aging was carried out in different solvents, such as n-hexane, methanol, and water, for different durations. The compression strength and compression modulus of the gel increased with increasing duration and the temperature of aging. Aging in water resulted in higher mechanical properties than aging in other solvents investigated. However, the specific compression strength and modulus of the samples aged in water declined remarkably.

Ionic liquids were also used in the synthesis of aerogels. It has been found that utilization of ionic liquid as reaction solvent along with long aging times (5 days–3 weeks) could result in stable aerogels. Although this is an elegant way of synthesizing aerogel, the removal of the ionic liquid requiring tedious extraction procedures brings an important disadvantage (Dai et al., 2000; Wu et al., 2012).

The classical synthesis route for highly porous silica aerogels usually requires supercritical drying to prevent the collapse of the delicate gel network upon the extraction of pore-filling liquid, as previously mentioned. Although the capillary forces approach zero at the supercritical state, shrinkage could still be observed. Heat treatment of silica gels in water has been investigated by Hæreid et al. (1995), who showed that both shear modulus increased with increasing aging time of silica gels in water at 40, 70, and 100 °C for various aging times up to 132 h and reached a maximum independent of the aging temperature. The increase in the aging temperature decreased the time required to reach the highest shear modulus. The surface area was found to decrease with aging time. The rate of the decrease was faster at higher aging temperatures. A correlation was found between shear modulus with respect to the surface area, showing a maximum in the modulus at a certain surface area. Along this line, Reichenauer (2004) showed that heat treatment of the wet silica gel in water at 60°C–80°C also resulted in decreased surface area and bulk density but improved mechanical properties while hindering the shrinkage during supercritical drying as compared to aging performed without heat treatment. He et al. (2009) developed a modified aging process for the synthesis of silica aerogels. In order to increase the porosity and improve the monolithic structure of silica aerogel, two methods were employed for the aging of silica gels derived from TEOS by a two-step sol–gel process where aging in 100°C-autoclave with TEOS/ethanol mixed solution and in pure ethanol at room temperature were carried out. The structural characteristics and physical properties of the two kinds of aerogels synthesized after supercritical drying were investigated and compared. Aging at 100°C in an autoclave resulted in silica aerogel with high pore size and pore volume, twice of that aged in ethanol at room temperature. High aging temperature and pressure can promote the dissolution and reprecipitation process of silica and the esterification of silanols, which improved the backbone strength of silica gel and hence formed silica aerogel with low bulk density and good monolithic structure, whereas the second aging route resulted in silica aerogel with high bulk density and presence of cracks.

Iswar et al. (2017) investigated the effect of aging on the physico-chemical properties of silica aerogel. Their results pointed out via dynamic oscillatory rheological measurements that aging within the gelation liquid reinforced alcogels, more prominently at high strain. Surface area decreased with increasing aging time and temperature as a consequence of the Oswald ripening process during aging. With increasing aging time and temperature, the linear shrinkage and bulk density decreased, and the pore size and pore volume increased for the ambient dried gels but remained nearly constant for supercritically dried gels. Gavillon and Budtova (2008) prepared a highly porous pure cellulose aerogel-like material and termed it as aerocellulose. The material was prepared from aqueous cellulose/NaOH solutions. The temperature and pH during the aging sequence greatly influenced the final properties of the material. Aerocellulose aged in water at ambient temperature showed a fibrillar structure, while aerocellulose regenerated at 70°C showed a cloudy structure in SEM images. As the aging bath acidity (H_2_SO_4_ was used) was increased, both the average pore diameter and the total porosity decreased.

Suh et al. (1999) investigated the effect of the aging process on the microstructure of gels, which were catalyzed by NH_4_OH and NH_4_F. Aging time and NH_4_F concentration affected the microstructure to a remarkable extent, that is higher aging times resulted in unimodal pore size distribution, and lower aging times usually resulted in bimodal distribution of pores.

Iswar et al. (2019) investigated the effects of aging on the microstructure and thermal conductivity of fiber-reinforced silica aerogel composites as an application for thermal insulation. Glass wool-silica gel composites were aged for varied times. Total porosity and thermal conductivity were virtually unchanged with respect to aging time if the gels were dried supercritically. Aging for much longer times (>8 h) improved (lowered) the thermal conductivity of atmospheric pressure dried gels, making their thermal conductivity similar to those dried using scCO_2_.

It is clear that among the reports in the literature, the effects of experimental factors used in the aging step are much more heavily investigated for the cases where APD was employed in order to develop stronger gels that would not collapse upon APD. However, there are only a scarce number of investigations on the effects of aging factors on gels dried supercritically, and more studies are needed in this area to develop aerogels with improved properties while taking into account the effects of the solvent exchange and drying on the final material performance.

### 3.2 Models related to aging step

It is very important to develop mathematical models that could describe the properties of the synthesized aerogels based on inputs such as the experimental conditions such as the ones described above for aging. Such models are very valuable to understanding synthesis-structure-performance relations and could be used to optimize and reduce the time and cost associated with some of the tedious experimental protocols. Along this line, Scherer et al. (1996) developed a model for predicting the shrinkage that occurs during the drying step based on the initial information on the initial pore size and modulus of the gel. The model was found to be in line with the experimental data for silica gels, given a number of aging treatments. Tamon and Ishizaka (1998) prepared RF aerogels via sol-gel polycondensation in a slightly basic aqueous solution followed by supercritical drying. The size and growth kinetics of polymeric species formed during the gelation and aging process was analyzed *in-situ* SAXS via applying Guinier and power-law kinetics. Data revealed that at the beginning of the RF hydrogel synthesis, clusters of 2 nm consisting of branched polymeric species formed, showing a mass fractal dimension. This was followed by the aggregation of the clusters, which formed particles around 3–6 nm with fractal surface. The hydrogel structure formed a gel around 4–7 nm, which was followed by aging. Upon aging, the surface of the networked particles became smooth without any fractals. More studies like this are required to better elucidate the nature of aging. Recently, Walker R et al. (2023) developed an information architecture, a silica aerogel graph database that included 10^3^ aerogels synthesized using different conditions, and a supervised machine learning neural network regression model to examine the synthesis process-aerogel property relationships. Machine learning models were used to further understand the influence of synthetic and processing conditions on aerogel surface area. The developed model maps from synthetic and processing conditions to predict the aerogel property and BET surface area. Surface area correlations were successful within an error of ∼109 m^2^/g, although the authors stated that with the addition of some validation experiments, the prediction error dropped to 5%.

## 4 Solvent exchange

One of the most crucial steps in the synthesis of aerogels is the solvent exchange, during which the pore liquid is exchanged with a solvent suitable for the subsequent drying method. During the process, not only the pore filling liquid is replaced, but also all unreacted monomers, oligomers, and the solvent that is used in the synthesis are removed (Schwan et al., 2021). The solvent exchange, which was previously described in Kistler’s initial work in the 1930s, is necessary to prepare intact, non-fractured aerogels at the end of the drying (Kistler, 1928). The drying of the wet gels without solvent exchange or the use of non-suitable solvents would result in fractured and shrunken aerogels due to the creation of high capillary pressures inside the pores (Şahin et al., 2017). One method to decrease these high capillary pressures is to select a solvent having a lower interfacial surface tension (Smith et al., 1992) and to replace pore filling liquid with this new solvent, thereby controlling how much the aerogel would shrink. The cause of this gel shrinkage being the most problematic part of the aerogel preparation has also been indicated by Kistler as the exchange of water to organic solvent and supercritical drying (Gurikov P et al., 2019).

Wet gels that are subjected to solvent exchange can either swell or shrink depending on the selection of different process parameters (Tripathi et al., 2018). It is known that especially organic aerogels shrink to a greater extent (Zhu et al., 2017) due to the formation of hydrogen bonds between the polymer strands, leading to a denser network. A higher density, in turn, would affect some properties such as pore volume, surface area, thermal conductivity, and many others. Thus, to obtain better physical properties, it is of utmost importance to understand shrinkage. The large shrinkage values obtained even with small changes in the solvent concentrations were also similar to the findings of Tanaka et al. (Tanaka et al., 1992; Gurikov P et al., 2019). They have observed that slight changes in the concentrations of acetone-water mixtures in which they were placing partially hydrolyzed acrylamide gels and crosslinked biopolymers, were resulting in discrete changes in the gel volumes (Tanaka et al., 1980). Thus, they have developed a model to semi-quantitatively explain phase transitions occurring in the preparation of those ionized gels. They interpreted the processes using the Flory-Huggins model (Tanaka et al., 1992). It is then reported by Gurikov et al. that this model can be used to qualitatively describe the shrinkage process in several ways (Gurikov P et al., 2019).

If supercritical drying is used, solvent exchange is especially important for the gels that are prepared in aqueous solutions or in solvents which are not soluble in scCO_2_ (Lebedev A. et al., 2021). Thus, they should be exchanged with more suitable solvents, commonly alcohols or acetone, with lower critical points and chemical activities to perform supercritical drying under more convenient and economic conditions (Subrahmanyam et al., 2015; Gurikov P et al., 2019). Moreover, the selected solvent should not destroy the structure of the wet gel by dissolving it or by any other means. In that regard, wet gels that are prepared by ionic cross-linking should be carefully considered. When those wet gels are subjected to the solvent exchange process, cross-linking ions, which are only physically bound to the matrix, can be washed out with the solvent, reducing the cross-linking degree (Raman et al., 2019). Ganesan and Ratke investigated this issue and demonstrated that the same K-carrageenan gels cross-linked with potassium thiocyanate behaved differently when subjected to either water first prior to solvent exchange with acetone or acetone alone (Ganesan and Ratke, 2014). The gel, which was primarily washed with water for 3 days, showed a significant shrinkage when it was subjected to acetone for solvent exchange. This was associated with the faster removal of cross-linking ions with water loosening the network strength.

Since Kistler’s time, the solvent exchange process has been carried out in the same manner by placing the synthesized wet gels in an excess new solvent (one step) or by performing a multistep procedure in which new solvent/water mixtures with increasing content of the solvent is used (Robitzer et al., 2008; García-González et al., 2011; Gurikov P et al., 2019). Although one-step solvent exchange seems to shorten the lengthy solvent exchange process, it usually causes a more drastic shrinkage (García-González et al., 2011; Subrahmanyam et al., 2015). That is why multi-step solvent exchange is commonly performed for various types of aerogels (García-González et al., 2011; Subrahmanyam et al., 2015). Using a mixture of solvents with water rather than the pure solvent reduces the concentration gradient acting on the gel, leading to gels more resistant to shrinkage. Thus, the addition of one or more intermediate steps in the solvent exchange with increasing concentrations of the solvent would end up with lower shrinkage. However, the reduction in shrinkage will also depend on the solvent concentration and the number of solvent exchange steps. Even with changing the solvent exchange period from four concentration steps to six concentration steps, the volume reduction was shown to reduce by 25%–30% (Mehling et al., 2009). Moreover, the use of lower initial concentrations of solvent resulted in reduced shrinkage even if it leads to a slower process (Subrahmanyam et al., 2015). On the other hand, carrying out a dynamic solvent exchange during which the solvent (acetone) was pumped continuously accelerated the solvent exchange process (Schwan et al., 2021). Moreover, the use of additional zeolites in the same bath to adsorb water kept the concentration gradients high, which increased the speed of diffusion and thus lowered the duration of the solvent exchange. Based on various factors affecting the solvent exchange, understanding its kinetics is a challenging task as the duration of the solvent exchange can vary significantly from hours to several days (Lebedev A. et al., 2021; Schwan et al., 2021). In that regard, Raman et al. employed a kinetic model to better describe and quantify the solvent exchange kinetics (Subrahmanyam et al., 2015). It was concluded that the concentration of the polymer and the cross-linking degree can affect the duration of the solvent exchange process. It was reported that the solvent exchange was fastest for low polymer concentration with a higher crosslinking degree. Halim et al. also investigated the effect of the solvent exchange duration on the final properties of silica aerogels derived from rice husk (Halim et al., 2021). It was found that performing solvent exchange for 9 days instead of 3 or 6 days led to the preparation of silica aerogels with an optimum BET surface area of 669 m^2^/g.

To reduce the time spent on the solvent exchange process, it can be combined with the supercritical drying in one apparatus (Lebedev A. et al., 2021). Instead of placing wet gels several times in pure or mixed solvents, the water inside the pores can be replaced with an organic solvent under pressurized CO_2_. The mass transfer under those conditions will be faster than atmospheric pressure, which in turn will reduce the duration of solvent exchange. Lebedev et al. developed a model to determine the parameters of the most efficient solvent exchange process under pressure (Lebedev A. et al., 2021). They used 2-propanol as the solvent and studied the ternary system “carbon dioxide-water-2 propanol”. Based on their theoretical study coupled with experimental work, they have established the necessary parameters to carry out the solvent exchange under pressure, reducing the time spent on the process, and thereby decreasing the cost of the aerogel production.

Not only the duration but also the temperature at which the solvent exchange is carried out can affect the quality of the prepared wet gels. Cardea et al. performed a solvent exchange at low temperature conditions (253 K) for chitosan aerogels (Cardea et al., 2010). They observed a lower shrinkage value associated with water-organic solvent (acetone) substitution at more stable gel conditions, namely, gelation conditions. Similarly, using a high washing temperature was found to crumble the silica gel network and led to large shrinkage values (Bangi et al., 2010). The effect of washing duration in addition to washing temperature was also investigated in the same study by Bangi et al. (Bangi et al., 2010). It was observed that increased durations lead to strengthened gels with less shrinkage. Furthermore, lower density aerogels were obtained with the use of lower chain alcohols such as methanol rather than ethanol, propanol, and butanol, which was partly associated with the lower surface tension of methanol.

**FIGURE 4:**
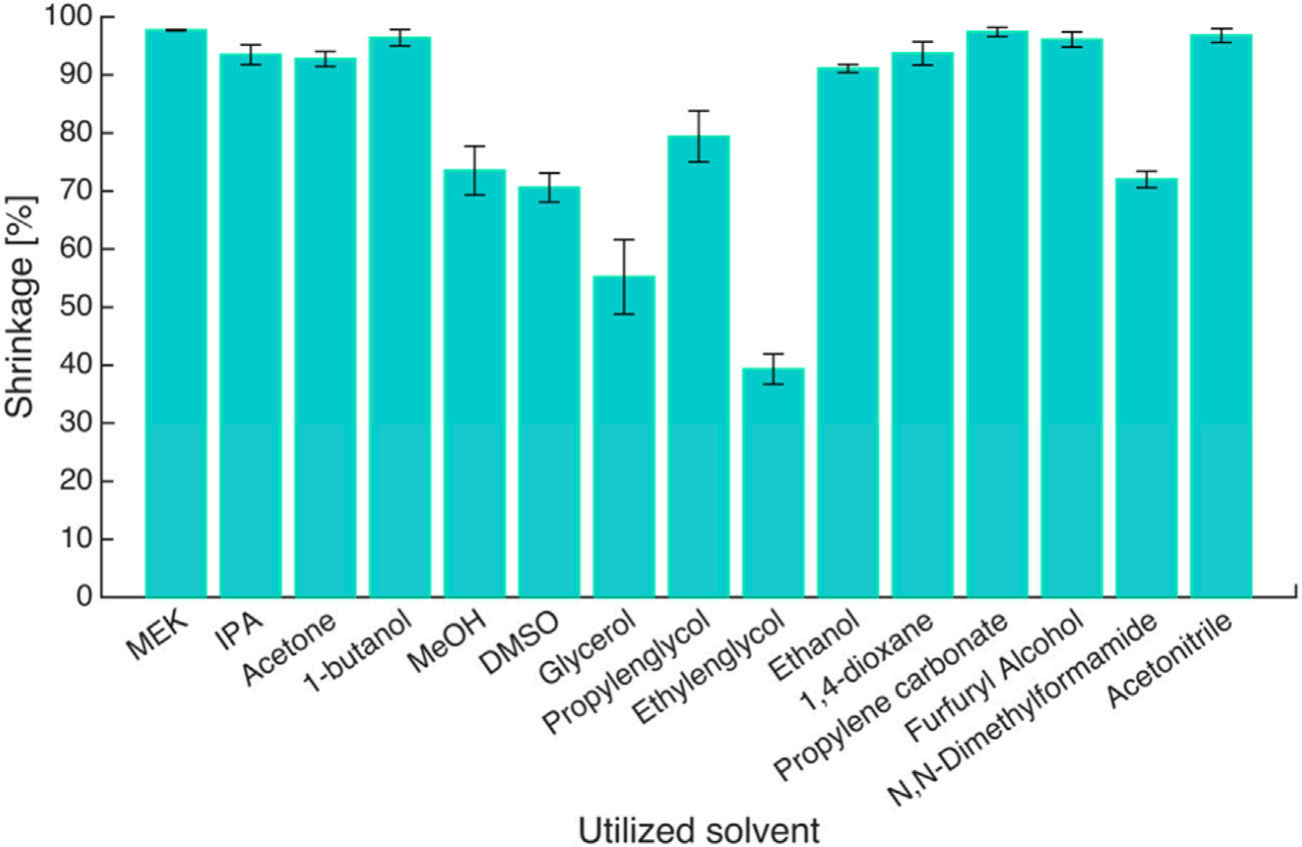
Volumetric shrinkage of gels in different solvents after one step solvent exchange. Reprinted from (Subrahmanyam et al., 2015) with permission, Copyright 2015 MDPI.

Considering those information, it can be said that there are various factors affecting the formation of the gel structure during the solvent exchange. Thus, a more detailed investigation is necessary to better understand the reasons for shrinkage during solvent exchange. It was suggested that the interaction of the polymer with the solvent might affect how much the volume of the wet gels will change. Subrahmanyam et al. tested 15 different solvents in the one-step solvent exchange process to explore their effect on the shrinkage of alginate aerogels (Subrahmanyam et al., 2015). Volumetric shrinkage was calculated for all resulting gels to quantify the effect of different solvents. The use of only 6 out of 15 solvents, such as methanol, DMSO, glycerol, propylene glycol, ethylene glycol, and DMF has resulted in volume shrinkage values of less than 90%, as shown in Figure 4.

However, it is already known that one-step solvent exchange would lead to extreme shrinkage, and the use of the same solvents in stepwise solvent exchange would result in more acceptable shrinkage values. These findings were further supported by the analysis of the difference in solubility parameters of polymers and solvents (Subrahmanyam et al., 2015). It was determined that the shrinkage of alginate gels was less when the solvent exchange process was performed, with solvents having higher solubility parameters. Similarly, Tripathi et al. investigated the relation between the solubility parameters and the volume change, and swelling of the wet gels during solvent exchange (Tripathi et al., 2018). They have furthermore proposed to use the solubility parameters to control the swelling behavior during solvent exchange in acetone-water mixtures of the cellulose acetate gels, which in turn lead to achieving aerogels with optimized properties. Another research group investigating the effect of different solvents on the gel shrinkage used 4 different organic solvents such as methanol, ethanol, isopropanol, and n-heptane, with a range of affinity for chitosan (Takeshita et al., 2020). Performing one-step solvent exchange processes has resulted mostly in broken or deformed aerogels. However, using primarily water-solvent mixtures prior to pure solvents decreased the large shrinkage values. They have also analyzed the relation between the macroscopic size and the microstructure of the gels and suggested that the structure of the cross-linked chitosan aerogels was based on the polymer-solvent affinity. When solvents with a higher affinity were used, the aerogel final structure was formed not during solvent exchange but during supercritical drying. Whereas when lower affinity solvents were used, the structure was formed during solvent exchange with a drastic shrinkage of the gel, which then endures supercritical drying.

Another group also reported that the volumetric yield of the wet gels can be associated with hydrogen bonding Hansen solubility parameter, which can be used for solvent selection purposes (Ghafar et al., 2017). Ghafar et al. further discussed the effect of the number of solvent exchange steps on the shrinkage of the wet gels based on the solvents’ nature (Ghafar et al., 2017). They measured the volumetric shrinkage of enzymatically crosslinked guar galactomannan wet gels performing one-step solvent exchange steps with 13 different solvents. Low volumetric shrinkage was observed when high polarity solvents were used for one-step solvent exchange. It was explained that polysaccharides tend to aggregate when they are placed in non-polar solvents as they will have a higher affinity towards neighboring polysaccharides than solvent molecules. The multi-step solvent exchange was further performed for the solvents, resulting in the lowest shrinkage (ethanol and DMSO) during the one-step solvent exchange. DMSO had better compatibility than ethanol during the process; however, the overall shrinkage after drying was similar with both solvents.

In some studies, it is also noted that the shrinkage is also size and shape-dependent (Ghafar et al., 2017; Gurikov P et al., 2019). The preparation of wet gels with smaller dimensions does not necessarily require the use of a stepwise solvent exchange process (Ghafar et al., 2017). Thus, it was suggested that when the concentration gradient between the outer surface and the center decreases, like in the case of spherical beads with small diameters, the shrinkage diminishes to a considerable degree (Gurikov P et al., 2019).

On the other hand, it is generally stated that biopolymer hydrogels that are prepared with higher polymer concentrations are more robust and resistant to shrinkage. It was likewise suggested that aerogels with less than 2 wt% polymer content would have larger densities than the others due to the higher shrinkage degree (Gurikov P et al., 2019). However, these findings rely mainly on the polymer nature, organization of polymer chains, and gel geometry. As an example, Dirauf et al. conducted a similar study observing the shrinkage of whey protein isolate aerogels with different protein content during one one-step solvent exchange process using ethanol (Dirauf et al., 2021). It was found that even the solvent exchange process of aerogels with less protein content takes less time; they had similar shrinkage values compared to high protein content aerogels.

Along the same lines, another strategy to reduce the shrinkage during solvent exchange would be to use additional precursors/monomers as reinforcements to obtain a stronger network. Although it seems to work well with silica gels, it is a rather difficult strategy to use for biopolymer gels (Gurikov P et al., 2019). In that regard, Einarsrud increased the stiffness and strength of wet silica gels by aging in monomer solutions, which then resulted in low shrinkage values during both ambient pressure and supercritical drying after washing (Einarsrud, 1998).

Alternative to reinforcing aerogels with the use of monomers, non-reacting agents, also called as fillers, can also be used (Gurikov P et al., 2019). In the study of Veronovski et al., starch was used as a filler to create a more stable alginate aerogel matrix (Veronovski et al., 2012). It was concluded that increasing the concentration of the filler was able to reduce the shrinkage after immersion in pure ethanol, even if the reduction was moderate. In the same study, it was also shown that using higher concentrations of the initial polymer resulted in a more stable crosslinked network. Moreover, the addition of a low molecular compound, a drug, during the synthesis also led to a reduced shrinkage for different initial concentrations of the polymer. It was suggested that the drug might be interacting with the polymer matrix, reinforcing the structure.

The preparation of composite polymer aerogels might also be given an alternative way of reinforcement to reduce shrinkage during solvent exchange. It was seen that the polymer networks can reinforce each other through hydrogen bonding and result in minor shrinkage during the solvent exchange process (Conzatti et al., 2017).

**FIGURE 5:**
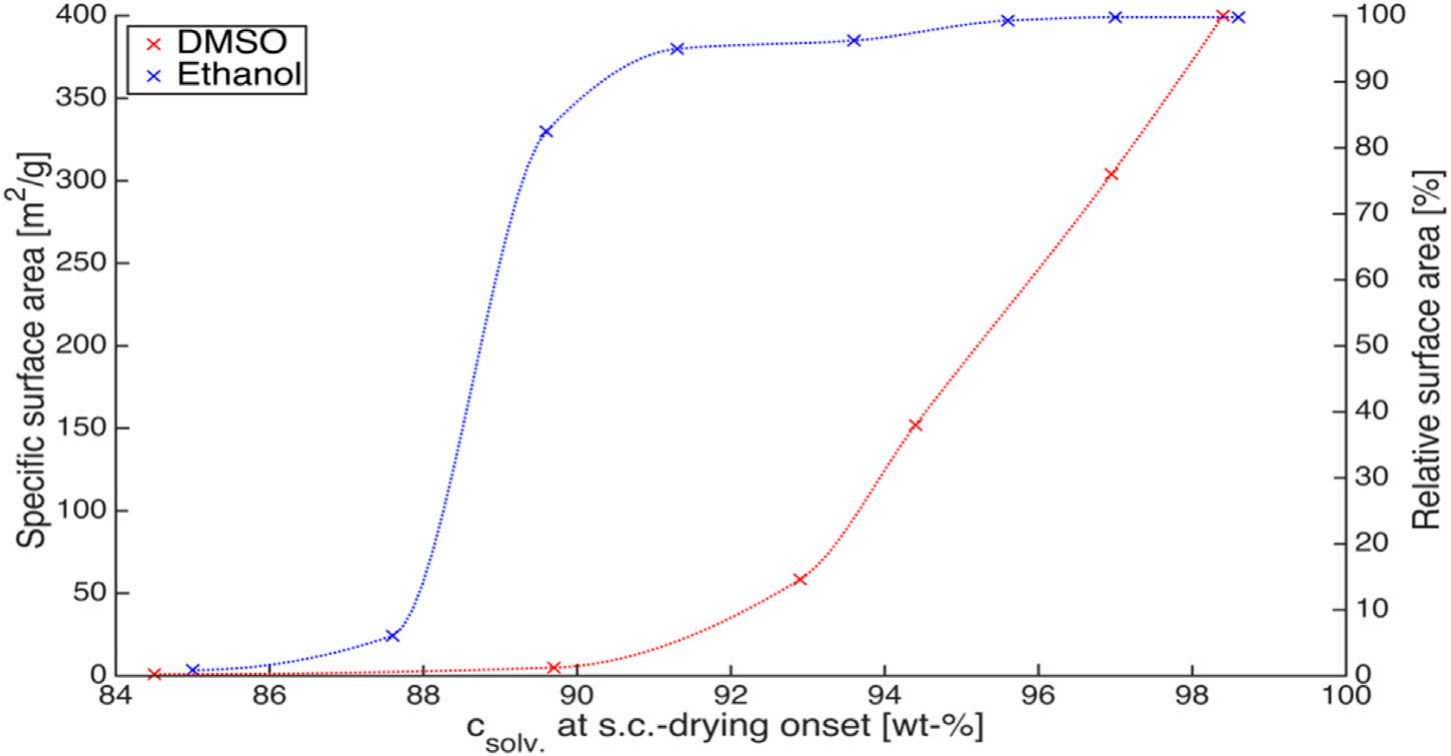
Change in the surface area with respect to solvent concentration achieved before supercritical CO_2_ drying for alginate aerogels. Reprinted from (Subrahmanyam et al., 2015) with permission, Copyright 2015 MDPI.

In conclusion, it is crucial to carefully select the solvent that will be used to wash the wet gels prior to drying. Another important point would be to know how much solvent exchange is necessary before drying the wet gels. In other words, it is critical to investigate the required concentration of the new solvent in the pores, considering both solvent recycling and final physical properties. In that regard, the analysis of the pore content was performed with Karl Fischer titration by Schwan et al. to understand the progress of the solvent exchange (Schwan et al., 2021). Furthermore, Subrahmanyam et al. have shown that alginate aerogels would retain their high surface area when drying is performed at ethanol concentrations higher than 93 wt% (Subrahmanyam et al., 2015). However, the concentration of DMSO required before drying the wet gels that are subjected to solvent exchange with DMSO was at least 98 wt%, as shown in Figure 5.

This effect should be further investigated to optimize the duration of the solvent exchange process. It would be advantageous to shorten the process; however, the interactions of the solvent with the solid matrix, its solubility in water and also in supercritical carbon dioxide, the number of steps, and the concentration of the solvent used should also be considered to minimize the shrinkage and to obtain an intact, non-fractured aerogel at the end of the drying.

## 5 Supercritical drying

Drying the wet gels is the final step of aerogel synthesis, which includes replacing the solvent filling the gel pores with air while the original gel structure is preserved. Removal of the solvent from the gel pores can be problematic since the capillary stresses in these pores, originating from the gas-liquid phase boundary, can be significant due to the nano-sized pores in the gel structure. These capillary stresses can lead to the destruction of the pores in terms of pore size and shape, collapse of the pores, and cracks in macroscopic gel structure (Bisson et al., 2003). Therefore, drying is a remarkably delicate step, where even slight variations in process conditions can result in the destruction of pores in the final product. There are three methods that can be used for drying the gels, including ambient drying, freeze drying, and supercritical drying (Şahin et al., 2017). Ambient drying is the evaporation of the solvent from the nano-sized pores at ambient pressure and temperatures ranging from room temperature up to 200°C, which is highly susceptible to making enormous changes in the gel structure and significant shrinkages. In fact, evaporation of the solvent from the pores causes remarkable increases in capillary pressure on the pore walls, which in turn leads to the destruction of the porous network. Using solvents with low surface tension or strengthening the gel network could be the solution for the problem of network destruction by capillary pressure, yet the capillary pressure would still be enough to damage the structure due to the nano size of the pores. Freeze drying is another method of drying the wet gels, during which the solvent is extracted from the pores by sublimation. This occurs after freezing the gels below the freezing point of the solvent, followed by decreasing the pressure below the sublimation pressure. After complete removal of the solvent, the system is brought back to the ambient condition. In this method, the destruction of the network may occur due to the stresses in the pore being applied during the freezing of the gels due to the growth of the solvent crystals, especially in the case of water, when it expands during the freezing. It can be further problematic when a solvent with an extremely low freezing point, such as ethanol, is used. Supercritical drying is an alternative method for drying wet gels where supercritical fluids (SCF) are used to extract the solvent from the pores of the gel network. In this method, the solvent, usually an alcohol, is first dissolved in the diffused SCF inside the pores, where a single-phase binary mixture is formed. Continuous flow of fresh SCF drains the solvent off the pores, leading to complete exchange of the solvent with SCF inside the pores. Subsequently, lowering the system pressure at temperatures above the critical temperature of the fluid leads to the removal of SCF from the pores without the formation of a phase boundary and huge capillary stresses, where the fluid phase inside the pores moves directly from supercritical to gas state without leaving any liquid solvent residue (Şahin et al., 2017; Leventis et al., 2002). Liquid-like density along with gas-like viscosity of SCFs enable them to be used as promising solvents in supercritical drying where zero surface tension of SCF and absence of a phase boundary during vessel depressurization significantly minimize the shrinkage and deformation of the gel network. Zero surface tension of SCFs not only avoids pore collapse but also permits better penetration and wetting of pores than liquid solvents do (Brunner, 2015). Furthermore, enhanced mass transfer characteristics due to higher diffusion coefficients in SCFs than liquids make supercritical drying an efficient and harmless gel drying technique, resulting in aerogel products with higher porosity and surface area than those dried with ambient and freeze-drying methods (Jin et al., 2004; Ciftci et al., 2017).

**FIGURE 6:**
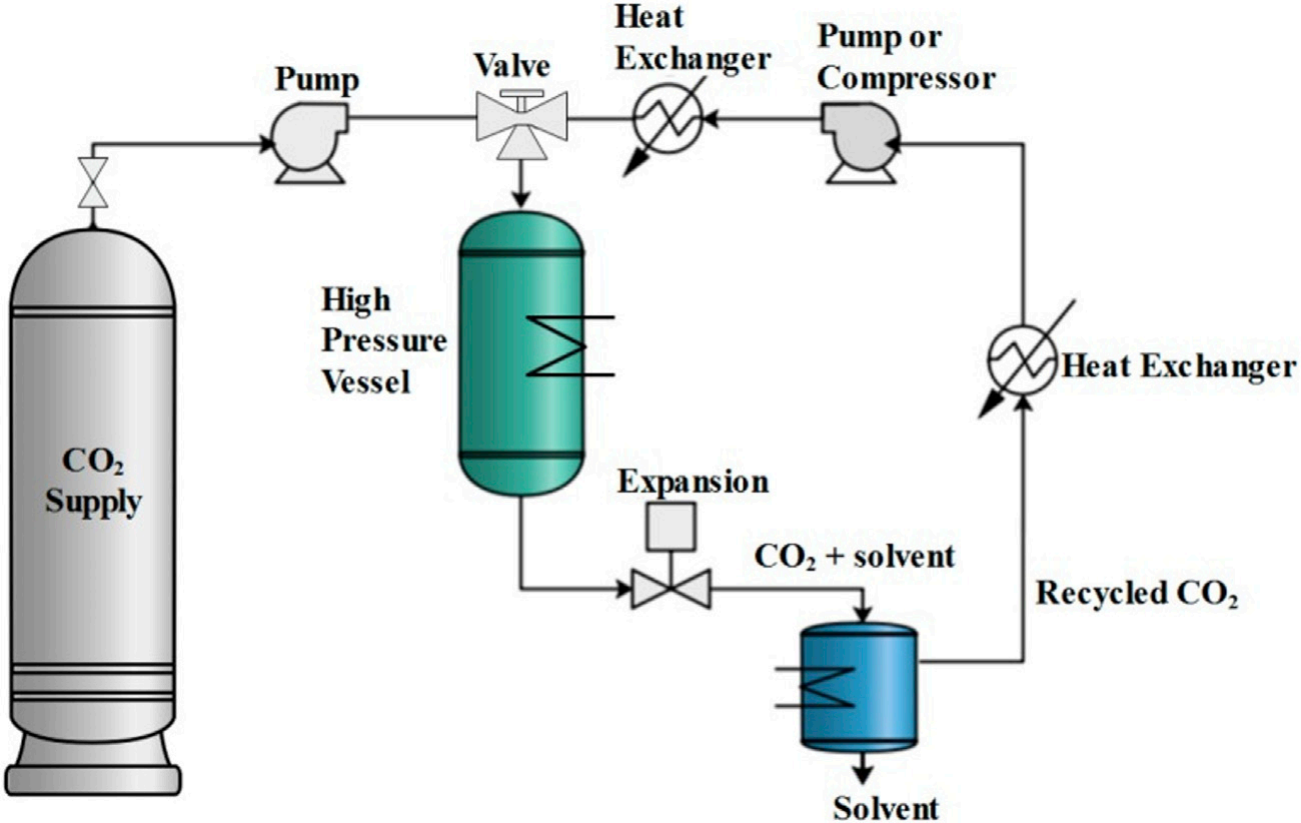
The schematic of a typical supercritical extraction apparatus. Reprinted from (Şahin et al., 2017) with permission, Copyright 2017 MDPI.

Supercritical drying is generally performed by a continuous flow of SCF, usually supercritical CO_2_ (scCO_2_), over wet gel for a certain period to obtain aerogels. Figure 6 shows the schematic of a typical supercritical extraction apparatus that can be used to dry the wet gels. The gels are first placed inside a high-pressure vessel filled with the parent solvent and heated above the critical temperature of the scCO_2_-solvent mixture. This is followed by pressurizing the vessel with the corresponding fluid up to the above critical pressure of the scCO_2_-solvent mixture. Subsequently, extraction of the solvent from the gel pores is followed by opening the outlet of the vessel and continuously charging the fresh fluid. The mixture of scCO_2_-solvent is finally flashed into a solvent collecting container at atmospheric pressure, which leads to the separation of CO_2_ rich gas phase and the solvent-rich liquid phase from each other. This step is continued until complete removal of the solvent from the gel, after which the vessel is discharged by closing the inlet valve and depressurization, during which the phase of CO_2_ inside the gel pores is changed from scCO_2_ to gas phase CO_2(g)_. Finally, dried gels are taken out of the vessel, after which air is gradually exchanged with CO_2(g)_ inside the pores.

ScCO_2_ is the most widely used SCF in supercritical extraction applications due to its favorable physical and chemical properties, especially its good solvation power for alcohols, which are the most used solvents for aerogel synthesis. scCO_2_ is particularly attractive due to its easily accessible critical point (T_c_ = 31°C, P_c_ = 7.38 MPa), being abundant, inexpensive, nonflammable, nontoxic, and environmentally benign. Furthermore, it is in the gaseous state at the ambient condition, which is one of the significant process advantages of scCO_2_ to obtain a completely solvent-free aerogels without the need to use any thermal or mechanical post-treatments. Using organic solvents in supercritical drying of aerogels presents significant drawbacks. The need for high temperatures (around 250 °C) and high pressure (5 ± 8 MPa) to achieve the supercritical state poses safety concerns. Additionally, the high temperatures can lead to rearrangement reactions in the gel network, altering the surface and causing pore filling and particle strengthening (Subrahmanyam et al., 2015). This results in aerogels with reduced specific surface area, less microporosity, and a more rigid structure.

Although the textural properties of aerogels, including specific surface area, porosity, pore volume and size, particle size, and shape (in the case of bead-formed gels), are formed during the gel preparation and aging steps, it is required to design a supercritical drying process with proper kinetics of drying at desired thermodynamic properties of the solvent-CO_2_ mixture to preserve the original textural properties of the wet gel. In this context, it is crucial to investigate the kinetic profile of supercritical gel drying, especially when designing systems based on aerogels. There are rare studies in the literature on the kinetics and effect of the process parameters of supercritical drying on the aerogel textural properties. Here, we reviewed some of them in this context.

**FIGURE 7:**
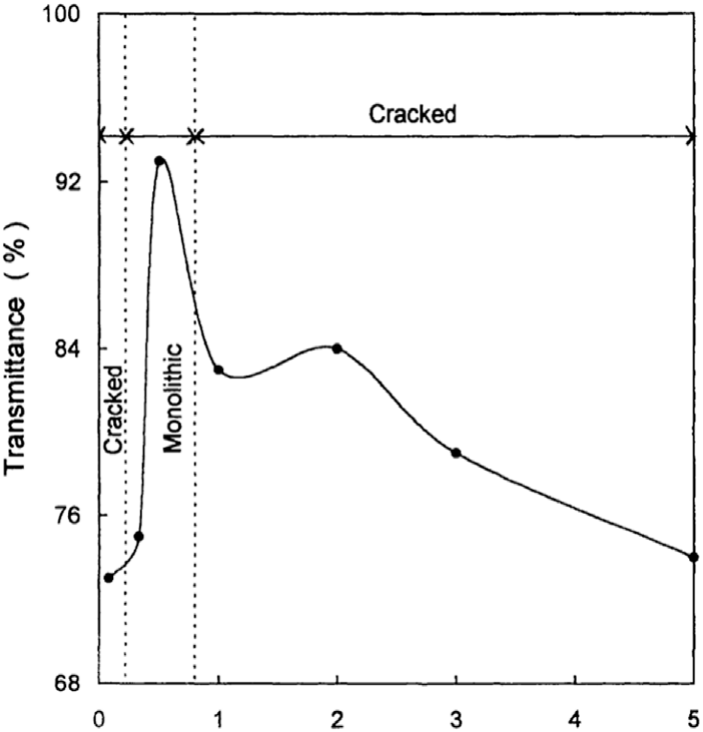
Variation of transparency and occurrence of cracks in monolith structure of silica with respect to the stabilization time (drying time). Reprinted with permission from (Venkateswara Rao et al., 1998) Copyright 1998 Taylor and Francis Group.

Earlier studies regarding the effect of supercritical drying on the aerogel’s characteristics focused on obtaining crack-free and transparent silica aerogels where the size of the gel sample all the drying and depressurization process conditions were shown as effective parameters on the textural properties of aerogels. Woignier et al. (Woignier and GeorgeScherer, 1994) conducted one of the earliest studies in the literature on the effect of depressurization rate as the final step of supercritical dying on the stresses developed in the gel network during this step. They showed that increasing the gel size and rate of depressurization leads to higher stresses, resulting in crack occurrence in the silica monoliths. The results were also in line with their developed theoretical model of the average stress under tested conditions where the calculated average stress of the crack-free samples was below the modulus of rupture (Woignier and GeorgeScherer, 1994). In another study, researchers demonstrated that most of the parameters of the drying process rely on an optimum range where the values out of upper and lower limits led to the formation of cracks in the silica aerogels. Venkateswara et al. (Venkateswara Rao et al., 1998) systematically investigated the effect of supercritical drying parameters (including the volume of excess alcohol (methanol), heating rate of drying autoclave, drying time, and depressurization rate) on the monolithicity and optical transmittance of the resulting aerogels. The results showed how much the textural characteristics of the aerogel are sensitive to the drying process conditions. As illustrated in Figure 7, it was observed that only a certain range of drying time between 0.2 up to 1 h led to a crack-free monolith of silica aerogel, a period during which the transparency of the aerogel showed a substantial fluctuation with a peak maximized at 93% transparency at 0.5 h of drying time. They also demonstrated the importance of other process conditions by determining the optimum range of the operating parameters to obtain crack-free and transparent monoliths (Venkateswara Rao et al., 1998). Novak et al. (Novak and Knez, 1997) also showed that the duration of the extraction affected the transparency and formation of cracks in cylindrical aerogel samples, where the wet alcogel pore filled with methanol was dried with scCO_2_. It was observed that the insufficient time of drying led to samples with non-transparent areas which were inside the gel with some cracks (in the worst cases). They also calculated the diffusion coefficient of methanol in CO_2_ by the theoretical model using the time of extraction and the geometry of the samples. In another study, Bommel et al. (van Bommel and de Haan, 1994) demonstrated that process temperature does not affect the time of drying significantly. They observed that crack-free monolith silica aerogels were obtained at a temperature of 35 °C thereby, there was no need to use high values such as 310 °C. It is worth mentioning that elevating the temperature also raised the pressure (from 35°C to 85 bar to 310°C and 150 bar). Thereby, according to the density diagram of scCO_2_ with respect to temperature and pressure (Yousefzadeh et al., 2022), this had minimal impact on the density and solvation capabilities of the supercritical fluid. As a result, it was reasonable to anticipate that the necessary drying time would not undergo significant alteration. Moreover, employing higher temperatures not only failed to increase the specific surface area of the dried samples but also led to the elevated vapor pressures of ethanol in the outlet, posing a challenge to the separation of CO_2_ and ethanol during the CO_2_ recycling process (van Bommel and de Haan, 1994).

Shafi et al. (Shafi et al., 2021) reported the effects of pressure and temperature on the drying process in supercritical drying of monolithic silica gels. The findings indicated that, at a constant temperature of 40°C, higher pressure leads to significantly reduced drying times. Specifically, at 40 °C and 25 MPa, a 60-min drying period suffices for complete ethanol removal from the silica aerogel monolith, while preserving the aerogel’s structure. Prolonging the drying time to 120 min shows no discernible improvement in the textural properties of the silica aerogel. This was probably due to the dramatically increasing density and solvation power of scCO_2_ by increasing pressure at 40°C, which is near the critical temperature of CO_2_. This, in consequence, resulted in an accelerated ethanol extraction rate. This approach demonstrated a promising solution to the prolonged drying times required for ethanol extraction from silica aerogels, especially in large-scale practical applications.

**FIGURE 8:**
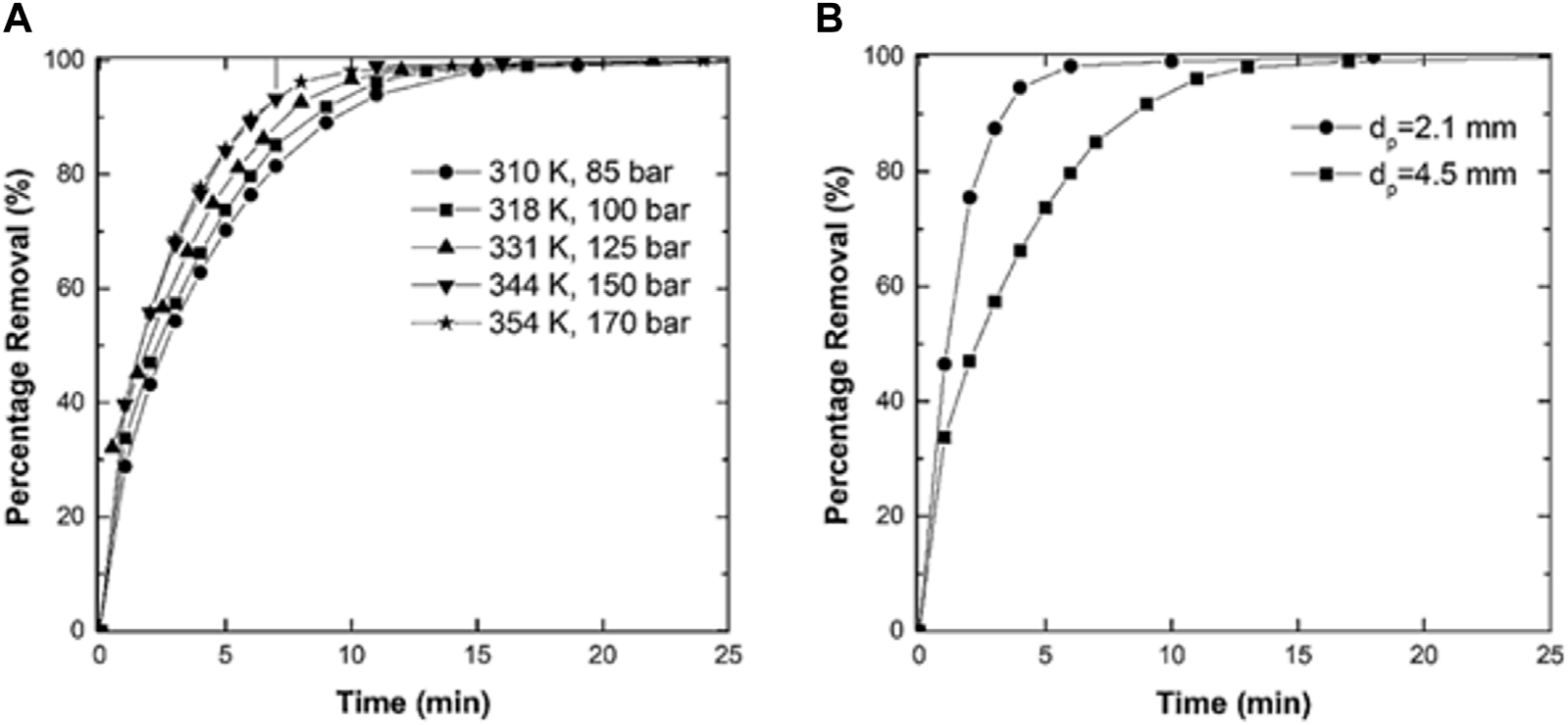
**(A)** percent removal of ethanol from the wet-gel for different temperatures and pressure for a constant density of inlet scCO_2_, **(B)** percent removal of ethanol from the wet-gel for different alcogel particle sizes. Reprinted from (Şahin et al. (2019a)), Copyright 2019, with permission from Elsevier.

Mathematical models were also studied to predict the kinetics and effect of the process parameters on the supercritical drying time of gels (Şahin et al., 2017). Some theoretical studies have been performed considering only diffusion as the mass transfer mechanism inside the gel, while others proposed that both diffusion and convective mass transfer are involved inside the gel during supercritical drying. Here, we first review some important modeling studies where only diffusion was considered inside the wet gel. The most studied model was conducted based on a two-way mass transport, diffusion of CO_2_ in and ethanol from pores. Mukhopadhyay and Rao (Mukhopadhyay and Rao, 2008) considered some main assumptions for their proposed mathematical model, which were used to describe the supercritical drying of silica alcogel. These assumptions were as follows. (i) cylindrical and equally accessible pores with the same radius and a rigid structure, one end closed and the other end open to scCO_2_. The wet and dried gels have matching pore size distributions. The gel’s pore configuration has been conceptualized using a parallel pore model, wherein pores of diverse dimensions run parallel and directly interact with scCO_2_ at the exposed end. Sometimes, pores could be considered in a series arrangement. (ii) Fick’s second law of diffusion applies to the dissolution of scCO_2_ in the pore liquid, involving both molecular and Knudsen diffusion, with the diffusivity of ethanol-scCO_2_ binary liquid phase depending on composition. (iii) The Peng – Robinson equation of state (P-R EOS) can be utilized to model the liquid volume expansion resulting from scCO_2_ dissolution. This allows for the determination of a mole fraction profile of scCO_2_ over time along the pore’s length. (iv) The convective transfer of ethanol vapor from the pore’s open end, aided by scCO_2_ flow, can be expressed using the Sherwood number correlation. It is assumed that the temperature of the liquid mixture remains consistent with that of scCO_2_. (v) The scCO_2_ flow rate can be regulated to ensure that the pore is consistently filled with a single phase until the pore boundary reaches the mixture critical mole (MCM) to avoid the occurrence of any condensation and formation of the liquid-vapor boundary layer during depressurization. They investigated the effects of gel thickness, temperature, pressure, and CO_2_ flow rate on the drying process at 313 K and 100 bar through simulations. The model proposed that CO_2_ diffusion into ethanol increased liquid volume, leading to excess liquid spilling from pores. This overflow was calculated by comparing volume changes between time intervals by employing Fick’s second law to study CO_2_ concentration over time and distance. The findings revealed that gel thickness and scCO_2_ flow rate had a significant influence on drying time. A lower flow rate notably extended the drying process, while lower temperatures and slightly higher pressures also increased drying time. Notably, the authors did not validate their results against experimental data. Orlović et al. (Orlović et al., 2005) also used similar mathematical models to predict the behavior of the supercritical drying of alumina/silica aerogels using experimental data. They demonstrated that models of parallel pores and pores in series were in good agreement with the experimental data. Simulations with the parallel pore model showed that drying time was significantly influenced by gel particle size and temperature. Smaller particles and higher temperatures can greatly reduce drying time. The effect of gel dimensions was also studied by Griffin et al. (2014), who investigated supercritical drying of cylindrical silica gel both experimentally and theoretically. They observed that reducing the thickness of gel samples was required to decrease the time of drying. Furthermore, increasing the CO_2_ flow rate above a certain value did not significantly affect the drying kinetics due to diffusion limited mechanism of solvent extraction from the pores, confirmed by the theoretical model where only molecular diffusion was considered into the effective diffusion coefficient within the gel media. Recently, Özbakir and Erkey (2015) conducted a study on comprehending the kinetics of supercritical drying in silica alcogel rods. Mass transfer mechanisms included diffusion within the alcogel, convection from its surface to the scCO_2_ stream, and convective mass transfer along the tubular vessel. Ethanol concentration was tracked in both phases over time and position. Experimental trials with continuous scCO_2_ flow around a cylindrical alcogel were conducted, measuring ethanol removal over time. The impact of CO_2_ flow rate, sample diameter, and effective diffusion coefficient on alcogel drying was explored through both experiments and simulations. The results revealed that increasing the flow rate of CO_2_ had little impact on extraction rates. On the other hand, it was observed that drying time increased with larger alcogel diameters but decreased with higher values of the effective diffusion coefficient. Şahin et al. (2019a) used the same model to study the drying kinetics in calcium alginate aerogels prepared in the form of spherical beads in a packed bed. Experiments were conducted at varying temperatures (35°C–81°C), pressures (85–200 bar), CO_2_ exit flow rates (2–4 L/min), and gel particle sizes (2.1–4.5 mm). Elevating the flow rate and increasing temperature reduced slightly drying time, while a more significant decrease in drying time was observed when smaller particles were used (Figure 8A, B). External mass transfer coefficients were calculated by developing a Sherwood number correlation by fitting the model to experimental data, yielding a satisfactory match between experimental and model results. They also conducted another study on the drying process of various M-alginate gel particles, where M represents Ca^2+^, Mn^2+^, Ni^2+^, Co^2+^, Cu^2+^, and Zn^2+^. The data showed an initial rapid removal rate followed by a slower pace as time advanced. The alignment between the model prediction and experimental kinetic data implied that the established mass transfer correlation for Ca-alginate particles also applies to the other M-alginate particles investigated in this study. Furthermore, it was observed that alterations in drying behavior among different gel types were primarily influenced by physical characteristics like particle size and porosity rather than the surface properties of the gel (Şahin et al., 2019b).

**FIGURE 9:**
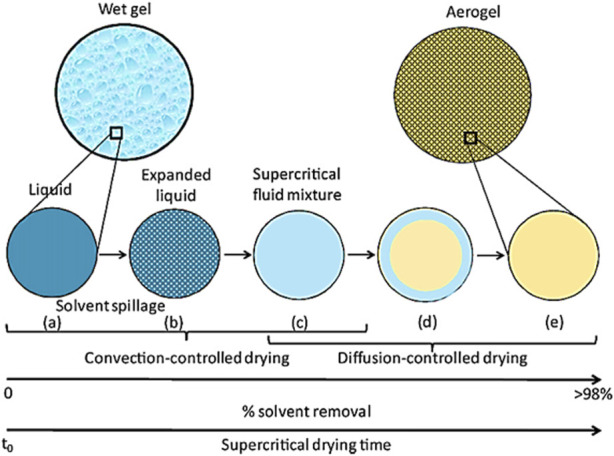
Two-step drying mechanisms of gels by supercritical drying technique combined convective and diffusion mechanisms. Reprinted from (García-González et al., 2011), Copyright 2012, with permission from Elsevier.

Some researchers have considered both convective and diffusion mechanisms of mass transfer inside the wet gel during supercritical drying. In a study conducted by Lazrag et al. (2018), two different approaches for the simulation of mass transfer during supercritical drying of cylindrical organogel. Two mathematical models were developed based on continuum mechanics to describe the CO_2_ flow dynamics and solvent mass transfer during drying. The first model treated the organogel as impenetrable, employing Fick diffusion for mass transfer. The second model considered the organogel as penetrable by CO_2_, combining convection with diffusion inside the gel particles. The results suggested that considering the organogel as a penetrable sample better aligned with the experimental data. Further simulations were performed to explore the impact of CO_2_ flow rate and gel thickness, indicating a significant influence of both parameters. Combined mass transfer mechanisms of convective and diffusion within the gel during the drying process had also been confirmed by experimentally and theoretically study by García-González et al. (2012), where the results revealed that considering only diffusion inside the gel failed to predict the experimental data for the solvent removal rate. They showed a schematic of mass transfer mechanisms inside the gel during supercritical drying by Figure 9, where a convection-controlled drying occurs at the initial moments of drying, which turns into diffusion-controlled drying after replacing a remarkable amount of solvent with the SCF and decreasing the concentration of the solvent inside the pores.

One of the large-scale supercritical drying studies was carried out by van Bommel and de Haan (1995). A method for producing large-scale monolithic silica aerogels through scCO_2_ drying was introduced. It was determined that achieving crack-free silica aerogel rods, plates, or tubes necessitates operating the system with an optimal design at approximately 35°C and 80 bar pressure. Economically comparing the production process of silica aerogel plates with different thicknesses and numbers of plates revealed that it was more cost-effective to manufacture three 1 cm thick plates rather than one 3 cm thick plate. Lebedev et al. (2015) introduced a mathematical model for simulating supercritical fluid flow, heat, and mass transfer in silica aerogel production in monolith form. The main aim was to use the model for scaling up the process, optimizing reactor geometry, and refining parameters for efficient production and desired aerogel properties on a large scale. The model was run using Ansys Fluent based on continuum mechanics. It considers the reactor’s interior as a compressible viscous fluid, described by the Peng-Robinson equation of state. The focus was on diffusive substance transport within the porous aerogel during supercritical drying. The experimental data was collected using a supercritical extraction apparatus with a 250 mL high-pressure cylindrical reactor, maximum working pressure of 300 bar, and working temperature of 100°C. The model was performed on two different areas, including the free volume of the reactor and inside the porous gel. In the former one, the convective and convection mechanisms for both mass and energy balance, along with the equation of motions, were solved. In the latter area, only molecular diffusion and convective heat transfer were assumed. The reactor wall was assumed to be at a constant temperature. The reactor was loaded with 10 monolithic gels. Their theoretical calculations were in good agreement with the experimental data. Thus, they used the mathematical model to scale up and optimize the process. This part of the study examined theoretically a 5-L apparatus geometry for supercritical drying. The proposed design was a cylindrical shape measuring 185 mm in both diameter and height. It features an elliptical bottom and a flat top. Positioned on the top are four inlets with a 4 mm diameter, while there is a single outlet at the bottom with a 7 mm diameter. The gel monoliths are situated on perforated shelves with varying numbers. Using this geometry and the developed model, the relationship between supercritical drying speed, reactor load and number of shelves, flow rate at various loads, and thickness of dried gels was demonstrated. The results demonstrated the significance of mathematical modeling for a cost-effective design of new equipment for processes like supercritical drying and for the transition from lab and pilot plants to industrial scale. The evaluation highlighted that increasing the number of shelves is more effective than increasing gel thickness. Additionally, it demonstrated that the initially selected CO2 flow rate suffices, and further increments do not significantly enhance process efficiency. Ultimately, utilizing this model for scaling supercritical drying proved to be a promising approach for creating and modernizing pilot or industrial units for such processes. Recently, the same research group performed a similar study on drying of alginate gel particles in an industrial 500 L reactor, where the effect of the system geometry on drying was investigated theoretically (Lebedev AE. et al., 2021). They showed that decreasing the number of shelves led to faster drying at the same loadings due to a decrease in stagnant volume inside. Furthermore, they recommended minimizing the number of shelves while keeping the thickness of the particles at possible minimum value. They also recommended the use of belt devices to apply a force movement of particles inside the apparatus, resulting in a reduced hydraulic resistance of the particles, which was recognized as an important factor in order to improve the process efficiency. In addition, it was concluded that it is better to increase the volume of the lateral by increasing the space between the shelf edge and the side wall and supplying the drying medium close to the apparatus tangent.

## 6 Carbonization

Aerogel carbonization is typically achieved through a pyrolysis process within the temperature range of 500°C–2,500 °C. During this carbonization process, hydrogen and oxygen groups are eliminated, forming a relatively pure carbon network (Araby et al., 2016). The pioneering carbonization of aerogels began with RF aerogel in 1989. RF aerogel was subjected to pyrolysis in a tube furnace at temperatures ranging from 600°C to 1100°C, under ambient pressure, and in an inert atmosphere. This process yielded carbon aerogel monoliths (Zuo et al., 2015).

The carbon matrix within carbon aerogels is composed of interconnected nanosized primary particles. Mesopores and macropores in carbon aerogels are attributed to the distance between these primary particles, while micropores develop within the primary particles themselves (White et al., 2014). Like other stages in the synthesis of carbon aerogels, carbonization conditions can be adjusted to fine-tune the morphological structure, including pore volume, pore size, and surface area (Allahbakhsh and Bahramian, 2015; Liu Y. et al., 2021; Abbas et al., 2022).

Carbonization can be controlled by four key parameters: temperature, heating rate, carbonization time, and the flow rate of sweeping gas. The choice of sweeping gas can also influence whether activation or doping occurs concurrently during carbonization. For example, introducing nitrogen (N_2_) into the carbon aerogel matrix can be achieved by using ammonia (NH_3_) during carbonization (Ünsal et al., 2019). The other parameters primarily impact the morphology of the resulting carbon aerogels, affecting factors such as surface area, pore volume, and pore size (Zhang et al., 1999; Xia et al., 2021).

Among these parameters, carbonization temperature has received extensive attention due to its significant impact on the structure of carbon aerogels. Varying temperature not only influences the morphology of the carbon aerogel matrix but also affects properties such as density (Zhang et al., 1999; Xia et al., 2021), electrical conductivity (Kim et al., 2005; Najeh et al., 2009; Zhou et al., 2021; Wang ML. et al., 2022), and thermal conductivity (Wang P. et al., 2022). Consequently, controlling carbonization conditions can have a positive influence on the performance of carbon aerogels in various applications, including electrochemical catalysis (Guilminot et al., 2007), insulation (Hu et al., 2019), and gas separation (Lee and Park, 2020).

This section will discuss the impact of the carbonization conditions on the physical properties of different carbon aerogel types.

### 6.1 Carbonization temperature

**FIGURE 10:**
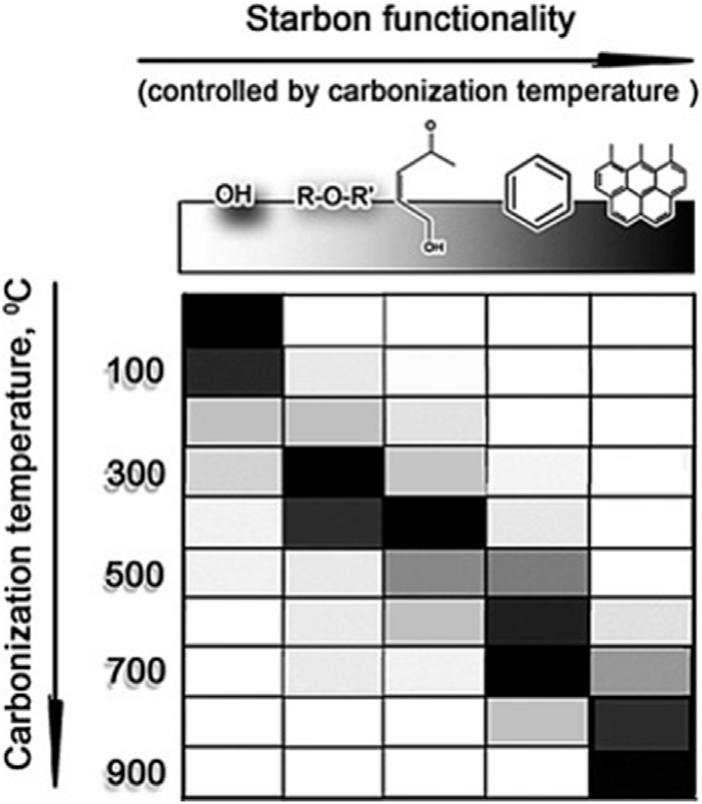
Distribution of Starbon functional groups as a function of carbonization temperature (color scale indicates relative amounts: black represents the highest amount). Reprinted with permission from (White et al., 2014), Copyright 2014, John Wiley and Sons.

The carbonization temperature plays a prominent role in shaping the elemental composition and chemical configurations within carbon aerogel structures. As temperatures increase, various organic groups undergo removal, transitioning into tar or gas occurs, consequently impacting the carbon matrix’s morphology and inherent nature. This transformation is illustrated in Figure 10 (White et al., 2014).

Characterization techniques such as Raman, XRD, and XPS have been employed to investigate the impact of carbonization temperature at an atomic level. The ID/IG intensity ratio, derived from Raman peaks, indicates the nature of the carbon aerogel matrix. It indicates the concentration of disordered carbon (defects) compared to ordered (graphitized) carbon (Hao et al., 2015). For instance, in chitosan-derived carbon aerogels with a hierarchical porous structure, the ID/IG ratio decreased from 0.83 to 0.73 as the temperature increased from 700°C to 900 °C. At higher temperatures (800°C and 900 °C), the appearance of the 2D peak in Raman spectra indicated a highly graphitic nature of the carbon aerogel structure (Hao et al., 2015). A similar trend was observed for cellulose nanofiber-derived aerogels, where the degree of graphitization increased with pyrolysis temperature (Long et al., 2021). However, it has been claimed that the ID/IG ratio may increase with pyrolysis temperature in the case of RF carbon aerogels due to matrix shrinkage, resulting in a loss of microporosity and imperfections in the carbon structure, thereby enhancing the D band intensity relative to the G band (Maldonado-Hódar et al., 2000).

XRD results corroborate these findings from Raman spectroscopy. Typically, XRD spectra exhibit two prominent peaks at 2-theta angles around 23° and 43°, corresponding to the (002) and (Raman et al., 2019) diffraction peaks of graphitic carbon (Hebalkar et al., 2005). In RF carbon aerogels, it has been observed that the peak around 43° becomes more dominant as the pyrolysis temperature increases. This indicates that the interplanar distance approaches the value for graphite (0.335), and the full width at half-maximum (FWHM) decreases, implying an elevated degree of graphitization with increasing carbonization temperature (Hebalkar et al., 2005; Wang SS. et al., 2020). This trend is similarly observed in chitosan-based carbon aerogels, where higher carbonization temperatures result in sharper (002) peaks (Lv et al., 2021; Xu, 2023).

XPS is another valuable tool for assessing the influence of carbonization temperature on carbon nature. The intensity of the C peak at 284.8 eV exhibits a direct correlation with pyrolysis temperature (Tian et al., 2021; Xie et al., 2021). This correlation aligns with the findings from Raman and XRD, highlighting the improved graphitization degree of carbon aerogels with increasing carbonization temperature. Additionally, the C/O atomic ratio rises as the graphitization degree increases with carbonization temperature (Bai et al., 2020; Tian et al., 2021; Xie et al., 2021).

XPS also aids in determining the atomic ratio of doped heteroatoms, such as N. Carbon aerogels derived from sources like chitosan naturally incorporate nitrogen atoms into their network, eliminating the need for an additional N-doping step. These nitrogen-doped carbon aerogels attract significant attention due to the enhanced performance conferred by nitrogen atoms in various applications such as electrolysis (Kobina Sam et al., 2020). Studies have shown that the N-content exhibits an inverse relationship with carbonization temperature after 600°C, which is the minimum temperature required to form chitosan-based carbon aerogels (Brovko et al., 2023). As carbonization temperatures rise, amorphous carbon is burned off, and carbon double bonds are formed with nitrogen-carbon (N-C) bonds. This results in the partial removal or transformation of pyridine and pyrrole configurations from the system, reducing the total weight percent of nitrogen (Wang ML. et al., 2022).

The most critical physical properties influenced by carbonization temperature are surface area, pore volume, and size. These properties can be determined through analysis of N_2_ adsorption-desorption isotherms of carbon aerogels. During carbonization, the release of gases creates various pores within the carbon matrix. In the context of typical N_2_ adsorption/desorption isotherms for carbon aerogels, a type-IV isotherm signifies the presence of pores with varying sizes, ranging from the micro-scale to the macro-scale (Lv et al., 2021). The sharp rise in the isotherm curve at the initial stage (P/P_0_ < 0.15) serves as compelling evidence for the existence of micropores (Hao et al., 2015). Additionally, the hysteresis observed between the adsorption and desorption branches indicates the presence of mesopores within the carbon matrix. Typically, to determine the distribution of pore sizes from these N_2_ adsorption/desorption isotherms, the BJH model is commonly employed (Mirzaeian and Hall, 2009; Hao et al., 2015; Lv et al., 2021; Tabrizi and Yavari, 2023). The number of pores increases within the carbon structure as a function of pyrolysis temperature until a point is reached where matrix shrinkage causes pores to collapse (Mirzaeian and Hall, 2009; Zhang et al., 2023). The optimal porosity for RF aerogels was found at 1073 K, resulting in the highest pore volume and surface area (Mirzaeian and Hall, 2009). This observation aligns with TGA analysis, which indicates that at 1073 K, no further mass loss occurs. Further increases in temperature cause shrinkage in the carbon network, blocking both micropores and mesopores and reducing the total pore volume (Maldonado-Hódar et al., 2000; Yamashita et al., 2003; Mirzaeian and Hall, 2009; Li L. et al., 2023). Table 1 summarizes the impact of carbonization temperature on the structural properties of carbon aerogels.

**TABLE 1:** Structure parameters of carbon aerogels as a function of carbonization temperature.

Carbon aerogel	Temperature (K)	S_BET_ (m^2^/g)	V_total_ (cm^3^/g)	V_micro_ (cm^3^/g)	V_meso_ (cm^3^/g)	%V_micro_	%V_meso_	D_avg_ (nm)	Ref
CRF001	600		0.187	0.1484	0.0386	79	21	2.33	Mirzaeian and Hall (2009)
	700	524	0.2874	0.2625	0.0249	91	9	2.27	
	800	669	0.38	0.33	0.05	86	14	2.19	
	1000	520	0.2905	0.2603	0.0302	89	11	2.23	
A-CFA	700	1016	0.55	0.46	-	84	-	2.14	Yang et al. (2018)
	800	978	0.50	0.34	-	68	-	2.47	
	900	1085	0.54	0.42	-	78	-	2.10	
	1000	1000	0.53	0.29	-	55	-	2.62	
CNT	600	492.7	0.607	0.188	0.418	31	69	4.93	Tabrizi and Yavari (2023)
	1050	203.7	0.298	0.086	0.212	29	71	5.86	
	1200	480.7	1.256	0.068	1.188	5	95	10.45	
CA	700	624.03	9	-	-	-	-	6.77	Wang et al. (2020b)
	800	677.65	9.28	-	-	-	-	6.33	
	900	696.22	1.03	-	-	-	-	7.24	
CA-4	550	709.42	0.39	0.31	-	79	-	2.19	Lv et al. (2021)
	600	634.78	0.34	0.27	-	79	-	2.17	
	650	611.86	0.35	0.27	-	77	-	2.29	
K	700	1524.3	0.56	0.53	0.3	95	54	-	Hao et al. (2015)
	800	2,435.2	1.09	0.62	0.24	57	22	-	
	900	2,209.4	1.49	0.15	1.13	10	76	-	
IPEC LSNa-CT	500	277.5	0.29	0.1	0.14	34	48	4.1	Brovko et al. (2019)
	600	438.3	0.43	0.18	0.25	42	58	4.3	
	700	395.2	0.41	0.18	0.23	44	56	4.1	
	800	394.9	0.42	0.17	0.25	40	60	4.2	
	900	331.6	0.37	0.14	0.23	38	62	4.4	
	1000	298.4	0.31	0.12	0.16	39	52	4.1	
Cas	400	800.7	-	0.454	-	-	-	-	Xie et al. (2021)
	800	830.5	-	0.491	-	-	-	-	
	1200	870.3	-	0.553	-	-	-	-	
A-RF	1000	470	-	-	0.628	-	-	-	Maldonado-Hódar et al. (2000)
	1400	28	-	-	0.639	-	-	-	
	1800	20	-	-	0.859	-	-	-	
COS	600	378	0.44	0.16	0.27	36	61	4.7	Brovko et al. (2023)
	900	460	0.37	0.23	0.14	62	38	3.2	
	1100	319	0.23	0.15	0.07	65	30	2.9	
	1300	113	0.1	0.05	0.05	50	50	3.7	
C/CA	1000	646.55	1.1					33.91	Li et al. (2023b)
	1200	449.61	0.8					47.79	
	1400	403.32	0.74					52.77	
	1600	297.1	0.6					70.43	
C/m-CNT-0	600	466.6	-	0.18	-	-	-	-	Wang et al. (2022b)
	800	546.69	-	0.25	-	-	-	-	
	1000	758.2	-	0.47	-	-	-	-	
C/m-CNT-0.05	600	493.67	-	0.52	-	-	-	-	
	800	552.34	-	0.68	-	-	-	-	
	1000	574.12	-	0.72	-	-	-	-	
RF-CA	800	532	-	0.22	-	-	-	-	Wiener et al. (2006)
	1000	571	-	0.24	-	-	-	-	
	1250	386	-	0.15	-	-	-	-	
	1500	267	-	0.09	-	-	-	-	
	1750	159	-	0.03	-	-	-	-	
	2000	133	-	0.01	-	-	-	-	
	2,250	120	-	0.01	-	-	-	-	
	2,500	123	-	0.01	-	-	-	-	
10-400-650CA	650	711.74	2.44	-	-	-	-	67.92	Wang et al. (2022c)
10-400-690CA	690	695.84	4.15	-	-	-	-	55.77	
10-400-750CA	750	775.06	2.61	-	-	-	-	65.41	
10-400-900CA	900	585.63	3.01	-	-	-	-	60.38	
10-400-1050CA	1050	790.19	1.26	-	-	-	-	2.54	
N3–650	650	676.24	0.353	0.031	-	9	-	3.271	Zhang et al. (2023)
N3–750	750	1038.02	0.426	0.089	-	21	-	1.742	
N3–850	850	903.26	0.413	0.053	-	13	-	3.254	
N3–950	950	856.33	0.402	0.047	-	12	-	2.568	
CA400	400	9.78	0.0470	-	-	-	-	8.1727	Qin et al. (2022)
CA600	600	29.84	0.0549	-	-	-	-	7.3461	
CA800	800	283.50	0.1300	-	-	-	-	5.5060	

In addition to N_2_ adsorption-desorption analysis, SEM and TEM images provide insights into pore size and distribution. SEM images reveal that the pore volume and surface area of PRF-CA increase with higher pyrolysis temperatures (Wang SS. et al., 2020). The PRF-CA carbonized at 900 °C had a honeycomb-like texture with larger open pores compared to those carbonized at 700°C and 800 °C. Consequently, it has the highest specific surface area, which was confirmed by the BET analysis. TEM images also offer evidence of the graphitization degree as a function of carbonization temperature, showing an increase in the graphitic portion of the carbon network with higher pyrolysis temperatures (Ma et al., 2022).

The relationship between carbonization temperature and the relative mass loss of carbon aerogels is well-established. As the temperature increases, there is a direct correlation with the reduction in the mass of carbon aerogels. However, it is important to note that this relationship eventually reaches a plateau, meaning that further increases in temperature no longer significantly impact mass loss (Xia et al., 2021). Regarding bulk density, the literature has presented conflicting findings, primarily due to variations in shrinkage percentages within carbon aerogels. For RF carbon aerogels, different studies have reported an increase (Hebalkar et al., 2005), a decrease (Zhang et al., 1999), or no significant impact (Kim et al., 2005) on bulk density as a function of carbonization temperature. For example, for cellulose carbon aerogels, two different values of bulk density were obtained when carbonized at 700°C and 950°C, with the aerogel pyrolyzed at the higher temperature having higher skeletal density but lower bulk density. This can be attributed to the greater shrinkage of cellulose microfibers at higher temperatures, leading to differences in densities (Meng et al., 2015).

The electrical conductivity of carbon aerogels exhibits a notable improvement with increasing temperature, eventually reaching a plateau (Kim et al., 2005; Najeh et al., 2009; Zhou et al., 2021; Wang ML. et al., 2022). As the carbonization temperature rises, there is a reduction in the organic residue, resulting in lower electrical resistance and, consequently, higher electrical conductivity. This inverse relationship between electrical resistance and carbonization temperature is well documented in the literature (Hebalkar et al., 2005; Zhou et al., 2021). The increase in electrical conductivity can be attributed to the greater amount of graphitization in the carbon network, a phenomenon supported by data from characterization techniques such as XPS and Raman, as discussed previously. The increase in the C1s and C/O ratio observed in the XPS analysis further validates this observation. Additionally, the intensified peak at 44° compared to the one at 23° in XRD indicates an increase in graphitization degree and, consequently, electrical conductivity. However, electrical capacitance does not necessarily follow the same trend as electrical conductivity. It is strongly influenced by the morphology of carbon aerogels. In some cases, such as cellulose fibers carbon aerogels, capacitance increases with carbonization temperature due to decreased micropores, which provide pathways for ions in the KOH electrolyte (Zhou et al., 2021). In contrast, studies on two different RF carbon aerogels showed that capacitance reached its maximum value at 800°C, with a subsequent reduction attributed to a decrease in surface area (Pekala et al., 1998; Kim et al., 2005).

The water contact angle of carbon aerogels is another property affected by pyrolysis temperature. An increase in carbonization temperature leads to a decline in hydrophilic functional groups on the surface of the carbon aerogel, resulting in an increased water contact angle (Song et al., 2020; Ibarra Torres et al., 2021). However, the contact angle remains relatively constant for carbon nanotube (CNT) aerogels as the carbonization temperature increases. This lack of a significant increase can be attributed to the contribution of surface roughness in determining the contact angle (Tabrizi and Yavari, 2023).

### 6.2 Carbonization heating rate

Pyrolysis mode represents additional factors that can fine-tune carbon aerogels’ properties. Tabrizi’s research delved into the impact of heating rate on CNT-doped carbon aerogels, exploring four different rates ranging from 5°C to 15°C/min (Tabrizi and Yavari, 2023). Interestingly, it was observed that the proportion of microporosity relative to the total surface area decreased with higher heating rates. An optimal condition emerged at a 7°C/min heating rate, maximizing the total surface area and pore volume (Tabrizi and Yavari, 2023). In another study, two distinct pyrolysis modes were compared (Brovko et al., 2017). Fast pyrolysis reached 1000°C within 30 min, while slow pyrolysis achieved the same temperature for chitosan-derived carbon aerogels over 15–16 h. The slow mode resulted in pore volume and micropore surface area values that were four and two times greater than the fast mode (Brovko et al., 2017). The distinction between the two modes lies in the carbonization mechanism. Fast pyrolysis involves rapid gasification and liquefaction of organic linkers, leading to a charring phase within the carbon network that poses insignificant obstacles for gas diffusion. This results in minimal changes in morphology. In contrast, slow pyrolysis promotes the diffusion of high-volatile linkers, generating micro- and mesopores within the carbon matrix (Brovko et al., 2017). It should be noted that the pyrolysis mode has received less attention in the literature than carbonization temperature, likely due to the latter’s more pronounced impact on carbon aerogel morphology. For instance, a study by Moreno et al. showed no significant difference in the pore structure of the carbon Xerogels as the heating rate was varied within the range (5°C–50°C/min) (Moreno et al., 2013).

### 6.3 Carbonization time

Carbonization time represents another adjustable parameter in the pyrolysis process. An investigation into boron-modified linear thermosetting phenolic resin (BPR) carbon aerogels explored the effects of carbonization time (30, 60, 90 min) (Liu H. et al., 2021). The results indicated a decrease in carbon aerogel particle size from 200 to 100 nm and an increase in pore size from 400 to 610 nm when the carbonization time increased from 30 to 60 min. However, extending the time to 90 min did not lead to significant changes in these values, which had already been obtained at 60 min and temperatures of 800°C and 1000 °C. TGA analysis revealed no substantial mass loss beyond 800°C, explaining why further increases in time did not notably impact the morphology of the carbon aerogel (Liu H. et al., 2021).

In studies where CO_2_ was used during pyrolysis to, etch carbon aerogels to increase their surface areas, time became a critical variable. For instance, increasing the pyrolysis time of RF carbon aerogels under a CO_2_ atmosphere from 0.5 to 2 h enhanced the ID/IG ratio from 1.7 to 2.3, signifying an improvement in graphitization degree (Abolghasemi Mahani et al., 2018). This prolonged pyrolysis time also enhanced the RF carbon aerogel’s porosity and surface area, with pore size distribution increasing from 1 nm to 4 nm (Abolghasemi Mahani et al., 2018). The additional time allowed for increased carbon burn-off, yielding a higher porous structure (Mirzaeian and Hall, 2009; Abolghasemi Mahani et al., 2018). Similar trends were observed when pyrolysis time was extended from 1 to 3 h for RF carbon aerogels with varying R/F ratios (Aghabararpour et al., 2019). The sample carbonized for 3 h at 800°C exhibited a higher surface area and electrical conductivity, while thermal stability remained independent of pyrolysis time, irrespective of the carbonization temperature used (Aghabararpour et al., 2019). Furthermore, subjecting carbon aerogel-polyamide (CAP) to a CO_2_ etching process for a duration of 3 h at a temperature of 1000°C, subsequent to a 5-h pyrolysis step under an Argon flow (150 mL/min) at 800°C, led to an enhancement in the presence of micro- and mesopores, consequently increasing the total surface area. Raman spectroscopy results demonstrated a reduced ID/IG ratio in comparison to CAP without CO_2_ etching, signifying a higher degree of graphitization and a reduction in structural disorders (Barim et al., 2022).

### 6.4 Alternative methods to produce carbon aerogels

Different techniques have been developed for producing various carbon aerogels, including carbon nanotube (CNT) aerogels and graphene aerogels, diverging from the conventional sol-gel method. These alternative methods encompass chemical vapor deposition (CVD), 3D printing, and ice templating, yielding distinct characteristics in the resulting carbon aerogels (Araby et al., 2016). CVD was utilized to produce lightweight carbon black aerogels (0.8 kg) in just 5 hours. These aerogels exhibit a low thermal conductivity of 0.049 W/(m⋅K) at 25°C, attributed to their low density, high porosity, and small pore size, which hinder phonon and radiation heat conduction (Zhou et al., 2023). Furthermore, CVD was used to prepare lightweight graphene/poly (dimethyl siloxane) composites with high electrical conductivity, reaching around 10 Scm^-1^. This conductivity is six orders of magnitude higher than chemically derived graphene-based composites. The absence of defect-related D bands in the composite signifies the high quality of graphene within the carbon matrix, contributing to superior electrical conductivity (Chen et al., 2011). Besides, porous carbon aerogels have been synthesized using direct ink writing and a combination of chemical techniques via 3D printing. These aerogels exhibit a high surface area and remarkable capacitance, reaching 148.6 Fg^–1^ at 5 mVs^–1^ (Yao et al., 2021).

Additionally, graphene oxide (GO) has been used as a precursor for synthetically synthesizing graphene aerogels through various routes (Singh et al., 2022). For instance, RF/GO composite carbon aerogel was produced via the sol-gel method by suspending GO in deionized water. The highest conductivity was achieved in aerogels with an RF: GO weight ratio of 4:1, two orders of magnitude higher than that of macroscopic 3D graphene networks prepared using physical cross-links (Worsley M et al., 2010). Another example is using L-cysteine (L-Cys) as both a templating and reducing agent for creating self-assembled graphene aerogels. The GO and L-Cys suspension solution was reduced in an oil bath at 90 °C for 3 hours, followed by freeze-drying to obtain the carbon aerogel. These aerogels exhibited exceptional adsorption capacity for organic dyes, such as methyl blue, primarily due to their hierarchical pore structure and large specific surface area (Zhang et al., 2015).

## 7 Conclusion and future directions

Aerogels have great potential for materials science and engineering breakthroughs due to their adjustable properties, such as pore sizes, specific surface areas, low thermal conductivities, and high sorption capacities. This review aims to enhance our understanding of aerogel production and changes in the conditions and parameters that impact aerogel properties during each production step to tailor the aerogel properties. Based on the studies mentioned above, the molecular structure and concentration of the precursor and solvent not only affect the gelation time but also the mechanical strength, volumetric shrinkage, bulk density, and pore sizes. The effects of catalyst type, concentration, and pH were discussed. Studies showed that catalysts can tailor the aerogel pore morphology by controlling the reaction rate and extent. The mechanism of aging and effective factors were explained. Despite the interest in biopolymer aerogels, aging has not been investigated widely. Solvent exchange is an important step in aerogel production since it generally causes aerogels to shrink, which leads to an increase in bulk density. Therefore, choosing a suitable solvent, concentration gradient, and duration is essential. In addition, the kinetics and process parameters of supercritical drying were discussed. For carbon aerogels, it was deduced that adjusting the carbonization temperature, heating rate, and time can tune the aerogel structure. Moreover, the developed models for each step of aerogel production were briefly presented.

To conclude, understanding the gelation mechanism, effects of synthesis conditions, and process parameters is fundamental to optimizing the aerogel properties, expanding the application area, and reducing aerogel production’s capital and operational costs. Therefore, further studies should focus on integrating advanced characterization techniques and computational modeling to tailor the aerogel properties precisely.
